# Insights into Genomic Epidemiology, Evolution, and Transmission Dynamics of Genotype VII of Class II Newcastle Disease Virus in China

**DOI:** 10.3390/pathogens9100837

**Published:** 2020-10-13

**Authors:** Bin Xiang, Libin Chen, Juncheng Cai, Jianpeng Liang, Qiuyan Lin, Chenggang Xu, Chan Ding, Ming Liao, Tao Ren

**Affiliations:** 1 College of Veterinary Medicine, South China Agricultural University, Guangzhou 510642, China; xiangbin@stu.scau.edu.cn (B.X.); chenlibin777@stu.scau.edu.cn (L.C.); caijuncheng@stu.scau.edu.cn (J.C.); 20171028006@stu.scau.edu.cn (J.L.); linqiuyan@scau.edu.cn (Q.L.); chgangxu@scau.edu.cn (C.X.); mliao@scau.edu.cn (M.L.); 2 Key Laboratory of Animal Vaccine Development, Ministry of Agriculture, Guangzhou 510642, China; 3 National and Regional Joint Engineering Laboratory for Medicament of Zoonosis Prevention and Control, Guangzhou 510642, China; 4 Key Laboratory of Zoonosis Prevention and Control of Guangdong Province, Guangzhou 510642, China; 5 Shanghai Veterinary Research Institute (SHVRI), Chinese Academy of Agricultural Sciences (CAAS), Shanghai 200241, China; shoveldeen@shvri.ac.cn

**Keywords:** Newcastle disease virus, genotype VII, Bayesian analysis, evolution, transmission, codon usage analysis

## Abstract

Newcastle disease virus (NDV) is distributed worldwide and has caused significant losses to the poultry industry. Almost all virulent NDV strains belong to class II, among which genotype VII is the predominant genotype in China. However, the molecular evolution and phylodynamics of class II genotype VII NDV strains in China remained largely unknown. In this study, we identified 13 virulent NDV including 11 genotype VII strains and 2 genotype IX strains, from clinical samples during 1997 to 2019. Combined NDV sequences submitted to GenBank, we investigate evolution, and transmission dynamics of class II NDVs in China, especially genotype VII strains. Our results revealed that East and South China have the most genotypic diversity of class II NDV, and East China might be the origin of genotype VII NDVs in China. In addition, genotype VII NDVs in China are presumably transmitted by chickens, as the virus was most prevalent in chickens. Furthermore, codon usage analysis revealed that the F genes of genotype VII NDVs have stronger adaptation in chickens, and six amino acids in this gene are found under positive selection via selection model analysis. Collectively, our results revealed the genetic diversity and evolutionary dynamics of genotype VII NDVs in China, providing important insights into the epidemiology of these viruses in China.

## 1. Introduction

Newcastle disease virus (NDV), a member of the genus *Orthoavulavirus* in the family *Paramyxoviridae* and the subfamily *Avulavirinae*, is the causative agent of Newcastle disease (ND), which causes huge economic losses to the poultry industry [1,2]. The genome of NDV is a single-stranded, negative-sense RNA that encodes six structural proteins, nucleoprotein (NP), phosphoprotein (P), matrix protein (M), fusion protein (F), hemagglutinin-neuraminidase (HN) and large protein (L). Two non-structural proteins, V and W, are generated due to an mRNA-editing event in the P gene mRNA [3].

NDVs are segregated into two groups, class I and class II, within a single serotype. With the exception of strains 9a5b and JS10-A10, all class I NDVs isolated to date are avirulent [4,5,6]. Majority of class II NDVs were consider to be virulent viruses based on the fusion cleavage site in fusion protein, responsible for most of the ND outbreaks reported to date [7]. Moreover, class II NDVs have a high degree of genetic diversity and could be classified into 20 genotypes (I to XXI, genotype XV contains only recombinant sequences and is excluded) [8]. Notably, genotype VII of class II NDVs caused the fourth ND pandemic, which continues today, affecting Asia, Africa, Europe and South America [7,9,10,11].

In China, the genotype VII NDVs were dominant circulating genotype, contributing to most ND outbreaks in poultry, while VIII, IX, and XII virulent NDVs were also occasionally reported [12,13,14]. Of note, the genotype VI virulent NDVs, referred to pigeon paramyxovirus type 1, also identified in China, inducing disease in pigeons, but show nonvirulence to chicken [15,16]. According to the updated classification criteria of NDV sub-genotypes—in which the cutoff value of the nucleotide distance was increased from 3% to 5%—all genotype VII NDV strains were divided into three groups, VII.1.1 (formerly VIIb, VIId, VIIe, VIIj, and VIIl), VII.1.2 (former VIIf), and VII.2 (former VIIh, VIIi, and VIIk) [8]. Previous studies have revealed the molecular characteristics and pathogenicity of NDV isolated from China [9,10,11,17,18,19,20]. However, the evolutionary dynamics of genotype VII NDVs and viral migration transmission across different hosts as well as regions in China, remained largely unknown.

Synonymous codons are not randomly chosen within and between genomes, a phenomenon referred to as codon usage bias [21,22], that allows viruses to efficiently survive and adapt to hosts [23,24]. Codon usage patterns are influenced by natural or translational selection and mutation pressure [25,26]. Most RNA viruses have low codon usage bias, which allows the viruses to replicate quickly and efficiently in the host cell by reducing competition with host genes [23,24,27]. Analysis of codon usage patterns in viral genomes provides insight into the fitness of a virus in different hosts [28]. Thus, the analysis of codon usage patterns of NDV could provide an insight to understand the NDV evolution.

In this study, we identified the complete F gene of 13 NDVs isolated from clinical samples, South China between 1997 and 2019. Combined NDV sequences in GenBank, we investigate the prevalence, molecular evolution, and phylogeography of NDVs in China, especially of genotype VII viruses. Moreover, we also explored the influence of mutational pressure and natural selection on codon usage patterns in the F gene of genotype VII NDVs in different hosts and estimated the contribution of wild and domestic birds to the dissemination and persistence of genotype VII NDVs. Our findings provide a comprehensive understanding of genotype VII NDVs in China.

## 2. Results

### 2.1. Virus Isolation and Identification

In this study, 13 NDVs were identified from clinical sample, Southern China, from 1997 to 2019. The complete F gene sequences of 13 NDVs were determined, including two strains of genotype IX (GenBank accession numbers: MT668582 and MT668583) and eleven strains of genotype VII (GenBank accession numbers: MT668584-MT668594) (Table S1).

### 2.2. Phylogenetic Analysis of Class II NDVs in China

**Figure 1:**
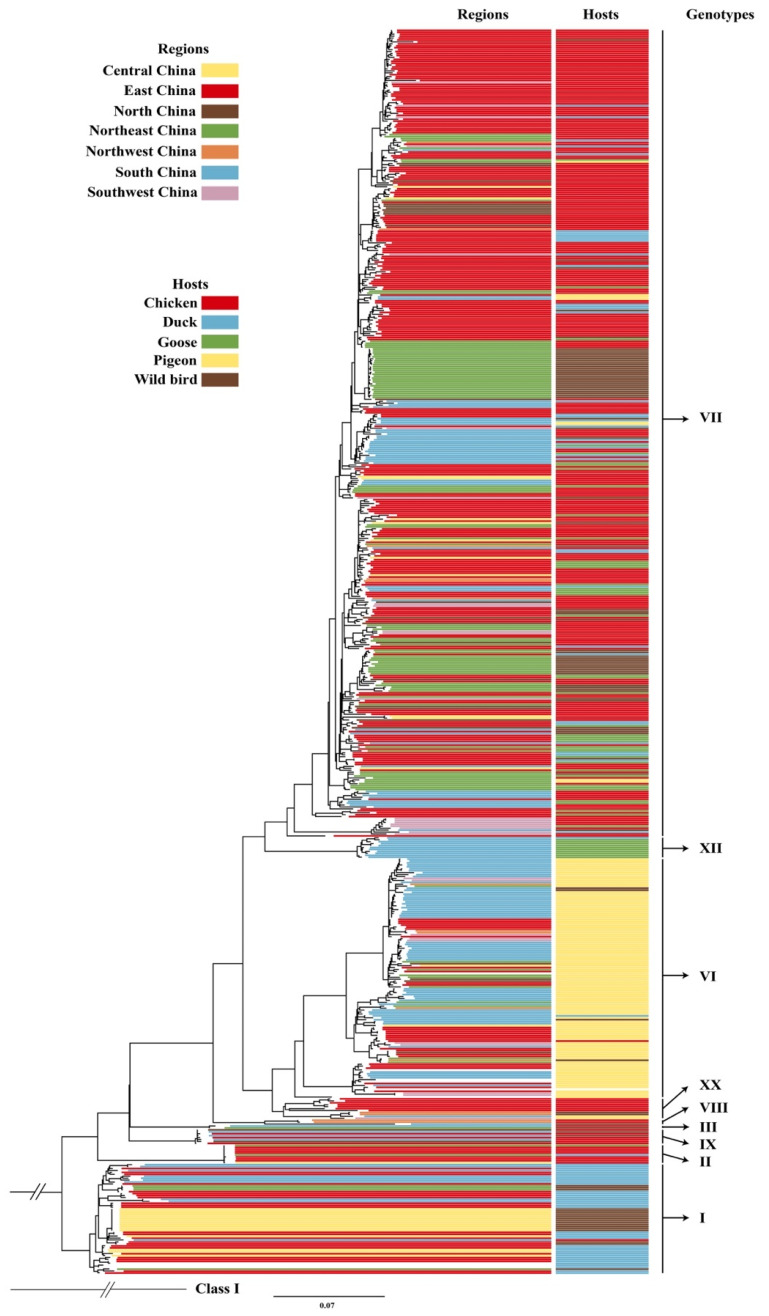
A maximum likelihood (ML) tree based on the F gene (≥1645 nt) of class II Newcastle disease virus (NDV) strains identified in China. The tree was constructed using IQ-TREE software with the GTR+F+R7 model. Colored lines indicate regions or hosts of origin.

To elicit information about the genotypes of class II NDVs in China, a maximum likelihood (ML) tree was constructed based on the F gene sequence (n = 645) of class II NDVs isolated in China from 1948 to 2019 (Figure 1).

**Figure 2:**
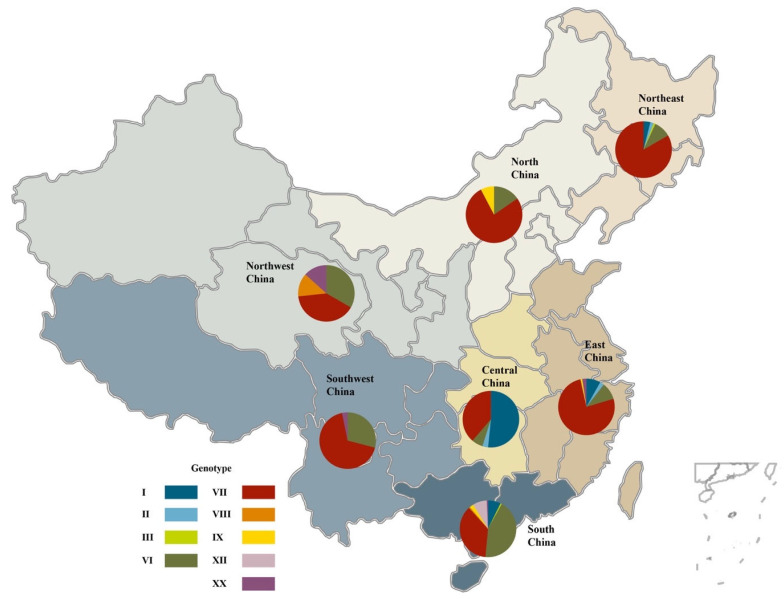
Distribution of class II Newcastle disease virus (NDV) strains isolated from China (submitted to GenBank before May 2020).

Based on the criteria detailed by Dimitrov et al. [8], the class II NDVs from China were grouped into nine genotypes, i.e., genotype I, II, III, VI, VII, VIII, IX, XII, and XX. Among these, genotype VII was the largest cluster (420/645, 65.1%), followed by genotype VI (125/645, 19.4%), I (57/645, 8.8%), XX (11/645, 1.7%), XII (11/645, 1.7%), II (10/645, 1.6%), IX (8/645, 1.2%), and VIII (3/645, 0.5%). East China had the largest number of class II NDV strains (295/645, 45.7%), followed by South China (136/645, 21.1%), Northeast China (102/645, 15.8%), Central China (31/645, 4.8%), Southwest China (31/645, 4.8%), North China (26/645, 4.0%), and Northwest China (15/645, 2.3%). East and South China had the greatest diversity of class II NDV genotypes (Figure 2).

### 2.3. Population and Evolutionary Dynamics of Class II NDVs in China

**Figure 3:**
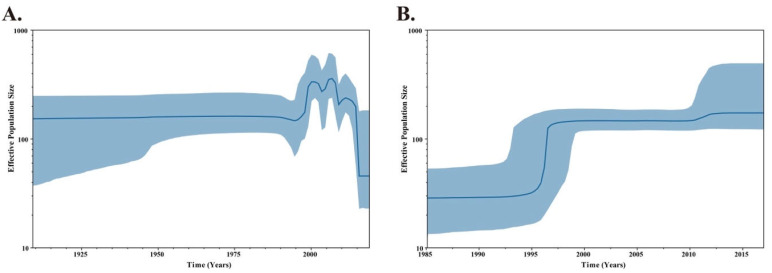
Bayesian skyline plot (BSP) of the F gene of NDVs. The dark blue line indicates the mean value of genetic diversity and light blue shading shows the 95% confidence interval. (**A**) BSP of class II NDV strains in China. (**B**) BSP of genotype VII NDV strains in China.

The demographic history of the class II NDVs genotypes included in this study was inferred using a Bayesian skyline plot (BSP) coalescent model, which plots changes in the pattern of effective population size through time, to examine the changes in genetic diversity during the migration of the virus. Figure 3A shows the result of the BSP analysis of class II NDVs in China. Initially, the population diversity of the class II NDVs exhibited a steady state. The effective population size of Chinese class II NDVs had increased and fluctuated between 2000 and 2015. After that, the genetic diversity of the NDVs decreased rapidly. By contrast, the effective population size of genotype VII NDVs remained essentially constant from 1985 to 1995 but increased rapidly around 1997. For the next decade, population diversity was again exhibiting a slight increase around 2010 (Figure 3B).

### 2.4. Geographical Migration of Genotype VII NDVs in China

**Figure 4:**
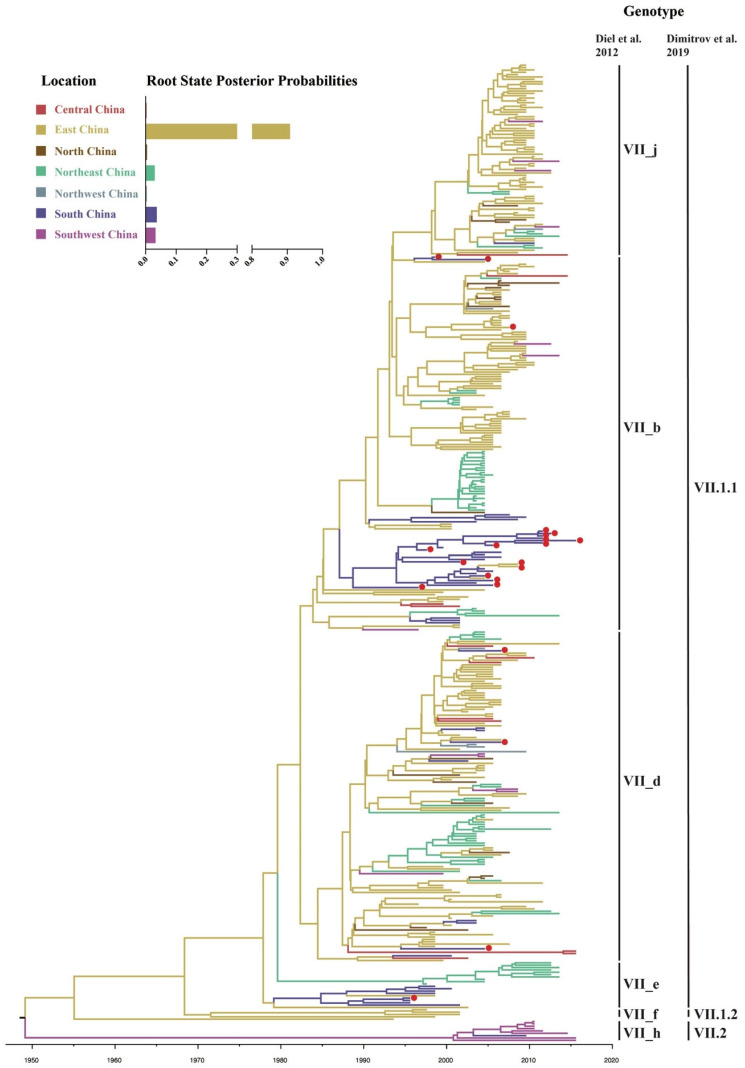
Maximum clade credibility (MCC) trees of the F gene (≥1645 nt) of genotype VII NDVs. The trees were constructed using BEAST version 1.10.4 (http://beast.community/). Twenty-two isolates identified by our laboratory are indicated by red circles. The root state posterior probabilities for regions are shown in the inset panel.

Because of its dominant role in the NDV epidemic in China, genotype VII was specifically targeted for follow-up analysis. A root-to-tip regression analysis of genotype VII NDVs showed a correlation coefficient and R^2^ of 0.6554 and 0.4295, respectively, confirming the presence of temporal structure. To estimate the geographic origin and the most significant epidemiological links of genotype VII NDVs in China, a discrete trait Bayesian phylogeographic method and a Bayesian Stochastic Search Variable Selection (BSSVS) procedure were implemented with different regions and hosts. The time-scaled maximum clade credibility (MCC) tree of genotype VII NDVs showed that these viruses isolated from China comprised three sub-genotypes, VII.1.1, VII.1.2, and VII.2, according to the recent classification system described by Dimitrov et al. [8] (Figure 4). Sub-genotype VII.1.1 was the dominant cluster from 1995 to 2019 (404/420, 96.2%) and contained sub-genotypes VII_b, VII_d, VII_e, and VII_j, which had been previously classified according to the criteria by Diel, et al. [29]. Our Bayesian phylogenetic analysis also showed that genotype VII probably firstly emerged in East China (root posterior probability = 0.91), with the time of most recent common ancestor (TMRCA) around 1951 (95% HPD: 1941–1960), well before the submission of the first genotype VII NDV sequence from China to GenBank (Figure 4).

**Figure 5:**
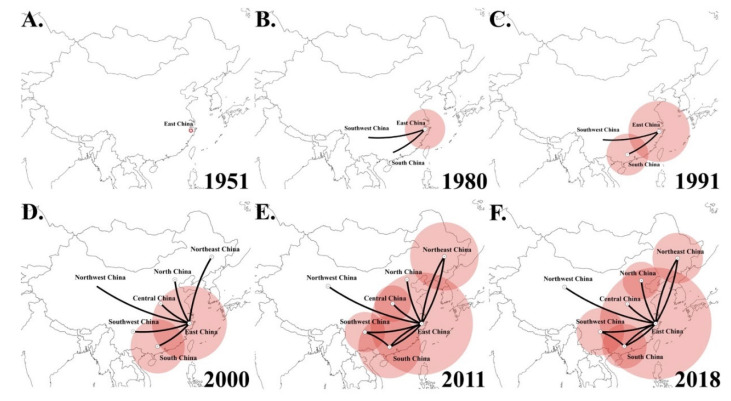
Spatiotemporal dynamics of genotype VII NDVs among different localities in China. Snapshots of dispersal patterns in (**A**) 1951, (**B**) 1980, (**C**) 1991, (**D**) 2000, (**E**) 2011, and (**F**) 2018. The data was collected from GenBank that submitted before May 2020 and this study.

The dispersal history of genotype VII NDVs was determined via globe animations using SpreaD3. Snapshots of dispersal patterns also showed that East China was the origin of genotype VII NDVs in the early 1950s (Figure 5A), and this genotype then propagated outwards over the next three decades (Figure 5B). The viruses had transmitted to South and Southwest China from East China by the early 1990s (Figure 5C), and by the early 2000s, genotype VII NDVs had spread throughout China (Figure 5D). Genotype VII NDV has been circulating among different regions in China (Figure 5E,F).

**Figure 6:**
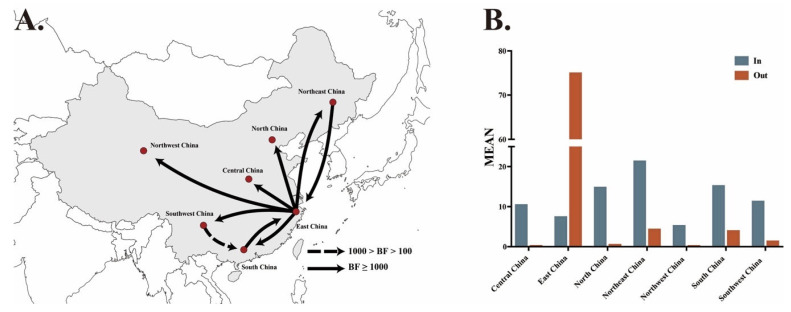
Spatial diffusion of genotype VII NDVs in China. Only statistically supported migrations with BF > 3 are shown. (**A**) Spatial diffusion pathways. (**B**) Histogram of the total number of state transitions.

A Bayesian phylogeographic analysis supported the presence of nine migration links in the diffusion of genotype VII NDVs in China (Figure 6A, Table 1). The highest mean rates were observed for migration from East China to Northeast China; the lowest mean rates were observed for migration from East China to Northwest China (Table 1). All the routes from East China to other regions had decisive support with high Bayes factor (BF) values, indicating that East China might play a key role in seeding genotype VII NDVs throughout China. This conclusion was further supported by the greater number of observed state changes supporting migration from East China than from other regions (Figure 6B). In addition, the routes from Northeast China to East China and from South China to East China also had decisive support (BF > 1000), and the route from Southwest to South had very strong support (BF = 111.96).

### 2.5. Host Dynamics of Genotype VII NDVs in China

**Figure 7:**
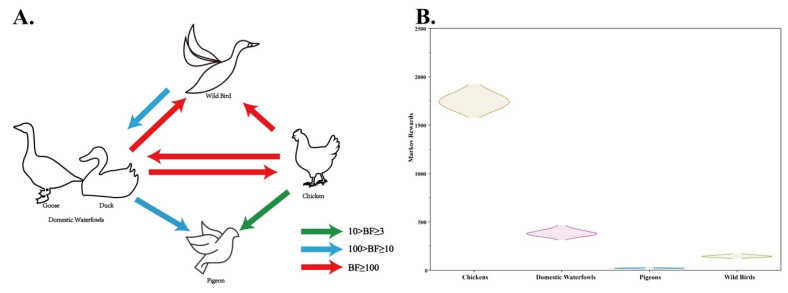
(**A**) Host transition of genotype VII NDVs in China. Only statistically supported migrations with BF > 3 are shown. (**B**) Density distribution of the total time spent in a particular host population (Markov rewards).

To estimate the transition of genotype VII NDVs between different birds in China, a BSSVS procedure was implemented by utilizing chickens, domestic waterfowl (ducks, and geese), pigeons, and wild birds as hosts (Table 2, Figure 7A). The viral transitions, from domestic waterfowl to wild birds and chickens, and from chickens to domestic waterfowl and wild birds, were identified as decisive support with high BF values (BF > 1000). Furthermore, the viral transitions from domestic waterfowl to pigeons (BF = 10.58) and from wild birds to domestic waterfowl (BF = 62.83) indicated strong support, while statistically significant support was identified from chickens to pigeons (BF = 6.11). The high mean rates of these transitions provided further evidence corroborating the results of BSSVS, suggesting that genotype VII NDVs in China were most likely transmitted from chickens or domestic waterfowl. The total Markov reward time was the highest in chickens (1746.55), followed by domestic waterfowl (386.61), wild birds (145.09), and pigeons (21.86) (Figure 7B), indicating that genotype VII NDVs were primarily sustained in chickens.

### 2.6. Codon Usage Pattern of F Genes in Genotype VII NDVs in China

Codon usage analysis was performed to further explore the mode of genotype VII NDV molecular evolution in China. Nucleotide and synonymous codon composition analyses showed that A (29.813 ± 0.631%) and U (25.662 ± 0.812%) were used more frequently than C (24.045 ± 0.903%) and G (21.510 ± 0.571%) in the F genes of genotype VII NDVs isolated from chickens. This tendency was similar for F genes of NDVs isolated from domestic ducks and geese. In addition, the nucleotide content of synonymous codons at the third position was A3s > U3s > C3s > G3s in chicken, duck and goose (Table S2). The relative synonymous codon usage (RSCU) value further confirmed that F gene codons ending in U/A were more frequent than those ending in C/G. Among the 18 preferred synonymous codons of the F gene, 15 ended with A or U, whereas only 3 ended with C or G. Analysis of codon over- and under-representation revealed that 4 out of the 18 preferred codons had RSCU values > 1.6; these codons were UCA (S), GCA (A), CAU (H), AGA (R). Additionally, the RSCU of F genes in genotype VII NDVs was essentially the same in chickens, domestic ducks, and domestic geese (Table S3).

**Figure 8:**
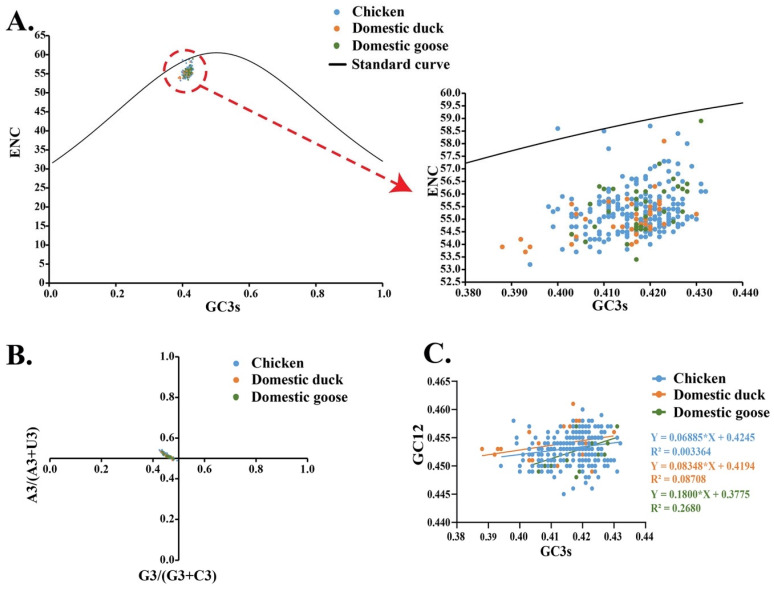
Codon usage pattern of F genes in genotype VII NDVs in China (**A**) Effective number of codons -plot analysis of the F gene of genotype VII NDVs, with ENC plotted against GC3s of different and hosts. The black line represents a standard curve showing when the codon usage bias is determined only by GC3s composition. (**B**) Parity Rule 2 (PR2)-bias plot [A3/(A3 + U3) against G3/(G3 + C3)] of the F genes of genotype VII NDVs. (**C**) Neutrality analysis (GC12 against GC3s) of the F genes of genotype VII NDVs.

Nearly all the data points were clustered below the standard curve in an effective number of codons (ENC) analysis based on different species, with only a few points located on or near the standard curve. If a genome lies on the standard curve (ENC expectation as a function of GC3s content), it indicates that codon usage bias is only affected by mutation pressure [30]. Hence, our ENC result indicates that factors other mutational pressure, such as natural selection were the dominant factors that influenced the codon usage bias (Figure 8A). In the parity rule 2 (PR2) plot, all points were separated from the center of the figure (both horizontal and vertical coordinates were 0.5), providing further evidence that codon usage bias was likely determined by multiple factors, including mutation pressure and natural selection (Figure 8B). Neutrality analysis revealed a narrow distribution and low GC3s values (0.432–0.388). To decipher the effects of mutational pressure and natural selection in different hosts, regression analyses were performed. We found the slopes of chicken group, domestic duck group, and domestic geese group were 0.06885, 0.08348, and 0.1800, respectively, indicating that the influence of mutation pressure on codon usage bias in the F gene was 6.89%, 8.35%, and 18.00% in chickens, domestic ducks, and domestic geese, respectively (Figure 8C).

The codon adaptation index (CAI) represents gene expression based on codon usage patterns, with higher CAI values (on a scale of 0 to 1.0) indicating better adaptation of a virus to its host [31]. The average CAI of F genes in genotype VII NDVs in chickens, domestic ducks and geese were 0.725, 0.659 and 0.653, respectively, indicating a better adaptation in chicken.

### 2.7. Positive Selection of the F Protein in Genotype VII NDVs in China

To understand the selection pressure acting on the F protein of genotype VII NDVs, an analysis of positive selection was performed. Six codons (sites 4, 8, 28, 121, 146, and 552) were found to be under positive selection in the F gene of genotype VII NDVs (Table 3). One site (121) was identified by three different methods and all the other sites were identified by all four methods. Among these, amino acid sites 4, 8, and 28 are located in the signal peptide, which marks the protein secretion pathway as well as the protein target location. Amino acid site 121 is located within the fusion peptide, and site 146 is located at heptad repeat A. These regions are involved in protein conformational changes that are crucial for viral infectivity and pathogenicity [32,33]. The amino acid at site 552 is located at the cytoplasmic tail.

## 3. Discussion

As a major infectious disease of poultry, ND outbreaks have been reported multiple regions of the world, including USA [34], Korea [35], and China [11], resulting in serious economic losses to the poultry industry. Since 1926, four global panzootics of ND have occurred. Genotype VII NDVs triggered the most recent fourth ND pandemic since 1980s, and was also responsible for most of ND outbreaks in China [9,11,17]. Since the isolation of the first genotype VII NDV strain in China in 1995, this genotype has gradually become the predominant NDV genotype in China [10,36,37,38]. Meanwhile, some virulent NDV in genotype III, VIII, IX, and XII were also sporadically identified in China [12,13,17]. The genotype VI virulent viruses mainly circulated in pigeons [39,40,41]. The genotype XII NDVs, which caused ND outbreaks in Peru and Vietnam, were emerged in waterfowls in Guangdong province in 2010 [12]. In our study, we found that a total of 65.1% (420/645) class II NDVs in China in GenBank with the addition of those viruses in current study, were classified into genotype VII. Of note, sub-genotype VII.1.1 was the predominant viruses (404/420, 96.2%), containing former sub-genotypes VIIb, VIId, VIIe, and VIIj, in line with the previous studies [11,18,38].

Phylodynamic models can be used to estimate the epidemiological and ecological characteristics of viral ancestors, allowing characterization and quantification of viral movements [42]. In this study, we conducted the first comprehensive phylodynamic study of genotype VII NDVs in China, indicating that East China was likely the origin of genotype VII NDVs in China. Further, TMRCA of genotype VII NDVs was dated to the year 1951 (95% HPD: 1941–1960), well before the first genotype VII NDV sequence from China was submitted to the GenBank. Hicks et al. [42] inferred that genotype VII NDVs migrated to South Asia, East Asia, and Africa from Southeast Asia and to the Middle East from East Asia, with TMRCA of 1940. A reasonable corollary to this would be that genotype VII NDVs emerged in South Asia in the 1940s and gradually spread to East Asia, including China. Over the next few decades, these viruses spread from East China to the rest of the country, becoming the dominant strains in the 2010s (Figure 5), and our results suggest that East China is likely the epicenter for the dissemination of genotype VII NDVs (Table 1, Figure 6). However, it is worth noting that some factors, such as sparse sampling, biased collection, and sequencing, could potentially affect this conclusion, especially considering the lack of epidemiological surveillance in the last century [41,43]. Therefore, wider and more sustained epidemiological surveillance in the future will be necessary to better understand the epidemiological and ecological characteristics of NDV, and is crucial for detecting genotype VII strains and ND outbreaks in general.

NDV shows a wide range of hosts, infecting over 250 bird species. Previous studies showed NDVs could be detected in fecal and oral swabs of birds, facilitating virus transmission [20]. Moreover, the genotype VII NDV strain, NDV/EG/CK/18/2015, could be transmitted from duck to chicken. Consistently, in the present study, the viral transitions of genotype VII NDVs between chickens and waterfowls with highly significant were observed. In China, small individual backyard farms with poor biosecurity where chickens and domestic waterfowls are free-range, inducing the frequent viral transmission between chickens and domestic waterfowls. Furthermore, a large number of live poultry markets existed in China, where domestic waterfowls and chickens from different regions were co-housed. Of note, high mean transition rate was from chickens and domestic waterfowl to wild birds, indicating NDV circulating in poultry could spill over into wild birds. This observation was supported by the early research [44,45,46,47]. Thus, to prevent the transmission among different birds, poultry management strategies should improve to avoid the direct and indirect contact of different poultry species and chilled instead of live poultry for sale should be adopted.

China’s poultry industry expanded dramatically in 1990s, but poor feeding conditions contributed to the spread of NDV, which could explain the significant increase in relative genetic density around 1995 (Figure 3). To control ND, biosecurity and vaccination has been employed in China [37,48], ND outbreaks have effectively been reduced in poultry farms. However, the widely used vaccine strain, LaSota, belonging to genotype II, is phylogenetically divergent from epidemic viruses (genotype VII) in China. Previous studies have demonstrated that NDV vaccines, phylogenetically more closely related to the epidemic viruses could provide better protection by the reduction of virus shedding [40,49]. Thus, genotype VII virulent NDVs still frequently identified in China after 2010. However, due to the development of intensive livestock farm with better biosafety, the number of small individual backyard farms decrease gradually. In addition, as genotype VII NDVs have received more attention as the dominant strains, other genotypes of class II NDVs have been isolated less and less. These could explain the sharp decline in the relative genetic density of class II NDVs after 2010, and a slight increase in the relative genetic density of genotype VII NDVs around 2010.

Codon usage bias analysis showed that the F genes of genotype VII NDVs exhibit low codon bias, consistent with the findings of a previous study [50]. RSCU, ENC-plot, and PR2 analyses suggested that the bias in F genes of genotype VII NDVs was influenced by mutation pressure and natural selection. Further, a neutrality plot analysis showed that natural selection was the more dominant of these factors. Low codon usage bias can be explained by the need of NDVs to efficiently replicate and survive in the host and to reduce the energy required for viral protein biosynthesis while avoiding competition with host protein synthesis [27]. Furthermore, natural selection can lead to weak codon usage bias while the virus is trying to adapt to the conditions within the host cells [51]. Moreover, CAI analysis suggested that the F genes of genotype VII NDVs are best adapted to chickens, domestic waterfowls (ducks and geese), and are better adapted to chickens than ducks and geese. This finding was consistent with our Bayesian analysis. In addition, we identified six amino acid sites under positive selection in the F gene of genotype VII NDVs; these sites might contribute to more efficient transmission among different bird species.

In conclusion, we isolated 11 genotype VII NDV strains from South China between 1997 and 2019. This study firstly revealed the genotype VII virulent NDV diffusion pattern among different host and geographic regions of China. Our results suggested that genotype VII NDV originated from East China and subsequently spread to South China and other regions, and chickens are the main hosts for genotype VII NDVs. Moreover, we also analyzed the codon usage pattern of F genes of genotype VII NDVs to provide a better understand the evolutionary changes of NDV. The findings of this study help us understand the underlying factors associated with genotype VII NDV evolution and viral diffusion among different hosts and regions in China.

## 4. Materials and Methods

### 4.1. Ethics Statement

Sampling procedures were performed in strict accordance with the guidelines of the South China Agricultural University Institutional Animal Care and Use Committee and were approved by the animal ethics committee of the South China Agricultural University, Guangzhou, China (permit number: 202020-V0223).

### 4.2. Surveillance and Sequencing

NDVs were isolated from clinical bird samples in Guangdong Province, South China from 1997 to 2019. Specimens were propagated in 10-day-old specific-pathogen-free (SPF) chicken embryos. The presence of NDVs was confirmed by hemagglutination and hemagglutination inhibition assays, and by reverse transcription polymerase chain reaction (RT-PCR) as described previously [12]. The primer sequences for amplification of F gene were F1 (5′- CAGGTDGCYAAGATACTCTGGAG -3′) and F2 (5′-GGCTCCTCTKACCGTTCTAC-3′). PCR products were cloned into pMD19-T and sequenced at Sangon Biotechnology (Guangzhou, China).

### 4.3. Collection of Datasets

All complete (n > 99% of the full-length open reading frame; n ≥ 1645 nucleotides, nt) F gene sequences from class II NDV collected in China and submitted to GenBank (NCBI, Bethesda, MD, USA) before May 2020 were downloaded and aligned using Multiple Alignment with Fast Fourier Transformation (MAFFT v7.221.3) [52]. Sequences determined to be 100% identical and originating from the same species, laboratory and/or outbreak, or with the identical name and date (retaining one representative in each duplicate pair/group) were omitted. After filtering, 632 sequences remained in the dataset, 409 of which were from genotype VII viruses.

### 4.4. Genetic and Phylogenetic Analyses

A ML tree was constructed based on F gene sequences in the curated dataset using IQ-TREE [53]; the initial tree was calculated using the GTR+F+R7 model and the final tree is supported by 1000 bootstrap replicates. To determine the temporal structure of NDV strains of China, a regression of root-to-tip genetic distance was performed using TempEst [54] based on the unrooted ML tree. To infer the evolutionary rate and timescale of NDVs, a Bayesian Markov Chain Monte Carlo (MCMC) simulation was performed using BEAST version 1.10.4 [55] with a strict clock model and constant size coalescent; the GTR+F+G4 substitution model was selected using ModelFinder with Bayesian information criteria [56]. The MCMC was run in parallel for six chains, each with 50 million steps and a burn-in of 10%; parallel result files were integrated via LogCombiner v1.10.4 (part of BEAST). Convergence of all parameters (i.e., effective sample sizes > 200) was confirmed visually using Tracer version 1.7 [57]. The MCC tree was inferred with TreeAnnotator (part of BEAST) and visualized via FigTree version 1.4.2 (http://tree.bio.ed.ac.uk/software/figtree/). The classification system for genotype VII NDVs in class II was based on the criteria reported by Dimitrov et al. [8]. Coalescent-based Bayesian skyline plots (BSP) were implemented to investigate the demographic history of class II genotype VII NDV strains in China using BEAST.

### 4.5. Bayesian Phylogeographic Analysis

To understand spatial diffusion and interspecies transmission patterns of genotype VII NDVs, an asymmetric continuous-time Markov chain phylogeographic model with the BSSVS [58] was implemented in BEAST. For this study, the Chinese landmass was divided into seven regions as per the traditional geographical divisions [47]. Genotype VII NDVs from different regions or different hosts were selected and coded as discrete states using a strict clock model and the constant size coalescent. BSSVS enabled the identification of the best supported individual transitions between the discrete states. The BF test was employed to identify significant non-zero transition rates and implanted in SpreaD3 version 0.9.7 [59], from which the visual animation file was exported. To confirm the reliability of the analysis, three independent BSSVS runs were performed. Significant transitions were determined based on the combination of both BF ≥ 3 and a mean indicator of 0.5; BF ≥ 1000 indicated decisive support, 100 ≤ BF < 1000 indicated very strong support, 10 ≤ BF < 100 indicated strong support, and 3 ≤ BF < 10 indicated statistically significant support. We also estimated the time spent for specific hosts (Markov rewards) via BEAST and Tracer.

### 4.6. Codon Usage Analysis

In order to understand the codon usage patterns in the evolution of genotype VII NDV, we investigated the codon usage bias patterns of NDVs from the three major host species, including chickens, ducks, and goose. The frequency of all nucleotides (A%, U%, G%, C%, AU%, GC%), the A, C, G, and U frequencies of codons at different sites (GC1%, GC2%, GC3%, GC12%, A3%, U3%, G3%, C3%, AU3%), and the CAI were calculated using the CAIcal SERVER (http://genomes.urv.cat/CAIcal/RCDI/) [60]. The nucleotide frequencies of synonymous codons at the third position (A3s%, U3s%, G3s%, C3s% and GCs%) and the ENC [61] were calculated using Codon W v1.4.2 (http://codonw.sourceforge.net/).

To find the most commonly used synonymous codons, the RSCU values for 59 codons were calculated using MEGA 7.0 (https://www.megasoftware.net/). RSCU values equal to, greater than, and less than 1.0 represented no bias, positive codon usage bias, and negative codon usage bias, respectively [62,63]. Codons with RSCU > 1.6 and < 0.6 were considered over- and under-represented, respectively [64]. Codon usage data for chicken (*Gallus gallus*), domestic duck (*Anas platyrhynchos*) and domestic goose (*Anser anser*) were obtained from the Codon Usage Database (http://www.kazusa.or.jp/codon/) [65]. The strains isolated from pigeon are too few to analysis effectively. And there are too many species of wild birds to select representative codon usage data for analysis.

A PR2 plot analysis was performed to investigate the impact of mutation and selection pressure on codon usage, with AU deviation [A3/(A3 + U3)] as the ordinate and GC deviation [G3/(G3 + C3)] as the abscissa. If A = U and G = C, the genome is evenly distributed in the center of the figure (both horizontal and vertical coordinates are 0.5), indicating that mutation pressure and selectivity (substitution rate) have the same effect on codon usage [66].

Neutrality analysis was performed to investigate the effect of natural selection and mutation pressure on codon usage bias. Using GC3s as the horizontal coordinate and GC12 as the vertical coordinate, the GC3s and GC12 contents of F genes in the dataset were plotted and a regression line was prepared. Regression lines with a slope close to 1 indicate that the genome is distributed almost diagonally, meaning that codon usage bias is only affected by mutation pressure; a decrease in the slope indicates an increase in the effect of natural selection [23,67].

### 4.7. Selection Model Analysis

To detect the selection acting on the F gene, an ML tree was constructed using Datamonkey (http://www.datamonkey.org/). The methods used to investigate positive amino acids sites included Single Likelihood Ancestor Counting (SLAC), Fixed Effects Likelihood (FEL), Mixed Effects Model of Evolution (MEME), and Fast Unconstrained Bayesian AppRoximation for inferring selection (FUBAR) [68,69,70,71]. Sites were considered to be under positive selection only when at least two algorithms were satisfied (*p* < 0.1 in SLAC, *p* < 0.05 in FEL and MEME, *p* > 0.9 in FUBAR).

## Figures and Tables

**Table 1:** Statistically supported migration rates of genotype VII NDVs in China estimated from the F gene.

From	To	Mean Transition Rate	BF ^1^	Posterior Probability ^2^
Southwest	South	0.69	111.96	0.955
Northeast	East	1.11	4492.98	0.999
East	Northwest	0.61	7426.62	0.999
South	East	1.18	85,461.49	0.999
East	North	1.52	170,928.25	1
East	Northeast	2.21	170,928.25	1
East	South	1.51	170,928.25	1
East	Southwest	1.21	170,928.25	1
East	Central	1.13	170,928.25	1

^1^ Only statistically supported transitions with BF > 3 are shown. ^2^ Posterior probability > 0.5 indicates well-support viral transition.

**Table 2:** Statistically supported host transition rates of genotype VII NDVs in China estimated from the F gene.

From	To	Mean Transition Rate	BF ^1^	Posterior Probability ^2^
Chicken	Pigeon	0.49	6.11	0.731
Domestic waterfowls	Pigeon	0.77	10.58	0.825
Wild birds	Domestic waterfowls	0.47	62.83	0.965
Domestic waterfowls	Wild birds	1.16	18,219.02	0.999
Chicken	Domestic waterfowls	2.94	72,882.81	1
Chicken	Wild birds	0.94	72,882.81	1
Domestic waterfowls	Chicken	1.33	72,882.81	1

^1^ Only statistically supported transitions with BF > 3 are shown. ^2^ Posterior probability > 0.5 indicates well-support viral transition.

**Table 3:** Selection analysis of the genotype VII NDV F protein.

AA	FUBAR (Post.Pro)	FEL (*p*-Value)	MEME (*p*-Value)	SLAC (*p*-Value)
4	0.952	0.012	0.01	0.007
8	0.925	0.018	0.03	0.055
28	0.998	0.001	0	0.001
121	0.896	0.028	0.04	0.060
146	0.978	0.003	0	0.019
552	0.997	0.004	0	0.001

## Supplementary Materials

The following are available online at https://www.mdpi.com/2076-0817/9/10/837/s1, Table S1: Strains information isolated in this study. Table S2: Strain information and the composition of the codon parameters of F genes of genotype VII NDVs. Table S3: The RSCU value of 59 codons encoding 19 amino acids according to two hosts of NDV VII F gene. The preferred synonymous codons are shown in bold.
