# Supplementary material for: Insights into Genomic Epidemiology, Evolution, and Transmission Dynamics of Genotype VII of Class II Newcastle Disease Virus in China
Source: Pathogens. 2020 Oct 13;9(10):837. doi: 10.3390/pathogens9100837 (PMC7602024; doi:10.3390/pathogens9100837)
Supplement: Supplementary file 1 [file pathogens-09-00837-s001.pdf]

Table S1. Strains information isolated in this study.

No.	Accession No.	Date	Location	Host
1	MT668582	2013	Guangdong	Black swan
2	MT668583	2013	Guangdong	Black swan
3	MT668584	2017	Guangdong	Chicken
4	MT668585	2003	Guangdong	Chicken
5	MT668586	2007	Guangdong	Chicken
6	MT668587	2008	Guangdong	Chicken
7	MT668588	1999	Guangdong	Chicken
8	MT668589	2007	Guangdong	Chicken
9	MT668590	2007	Guangdong	Gadwall
10	MT668591	1998	Guangdong	Goose
11	MT668592	2013	Guangdong	Goose
12	MT668593	2013	Guangdong	Duck
13	MT668594	2013	Guangdong	Duck

Table S2. Strain information and the composition of the codon parameters of F genes of genotype VII NDVs.

Strain information	GC1%	GC2%	GC12%	GC3%	A%	C%	U%	G%	GC%	AU%	U3s	C3s	A3s	G3s	GC3s	ENC	CAI	A3%	C3%	U3%	G3%
AF358786_CH	46.800	43.000	44.900	42.400	29.723	22.623	26.233	21.420	44.043	55.957	0.338	0.264	0.357	0.241	0.410	55.100	0.726	28.700	22.563	28.881	19.856
DQ067447_CH	46.800	43.300	45.050	43.300	29.603	22.924	25.933	21.540	44.465	55.535	0.328	0.270	0.354	0.243	0.419	54.800	0.730	28.520	23.105	28.159	20.217
DQ227249_CH	47.500	43.100	45.300	43.100	29.723	23.045	25.692	21.540	44.585	55.415	0.323	0.274	0.362	0.238	0.418	55.600	0.726	29.242	23.466	27.617	19.675
DQ227253_CH	46.900	43.700	45.300	43.900	29.603	23.105	25.572	21.721	44.826	55.174	0.324	0.275	0.354	0.243	0.423	55.300	0.725	28.520	23.466	27.617	20.397
DQ228922_CH	47.500	43.300	45.400	43.000	29.844	23.165	25.572	21.420	44.585	55.415	0.323	0.274	0.364	0.235	0.416	55.100	0.724	29.422	23.466	27.617	19.495
DQ363530_CH	46.800	43.000	44.900	42.100	30.144	22.804	25.933	21.119	43.923	56.077	0.331	0.270	0.369	0.226	0.404	55.100	0.722	29.783	22.924	28.159	19.134
DQ363533_CH	46.800	43.100	44.950	42.100	30.084	22.804	25.933	21.179	43.983	56.017	0.331	0.269	0.369	0.226	0.404	54.700	0.722	29.783	22.924	28.159	19.134
DQ363535_CH	47.500	43.100	45.300	44.000	29.483	23.045	25.632	21.841	44.886	55.114	0.325	0.276	0.351	0.247	0.426	57.200	0.724	28.339	23.466	27.617	20.578
DQ363536_CH	47.100	43.100	45.100	43.900	29.603	23.045	25.692	21.661	44.705	55.295	0.327	0.276	0.352	0.246	0.424	56.900	0.724	28.339	23.466	27.798	20.397
DQ363537_CH	47.100	43.900	45.500	44.000	29.362	22.984	25.632	22.022	45.006	54.994	0.326	0.272	0.348	0.251	0.426	58.400	0.729	27.978	23.285	27.978	20.758
DQ485229_CH	47.300	44.200	45.750	43.000	29.422	22.864	25.752	21.961	44.826	55.174	0.328	0.261	0.355	0.250	0.417	55.200	0.724	28.881	22.383	28.159	20.578
DQ485231_CH	46.400	43.100	44.750	43.700	30.084	23.105	25.511	21.300	44.404	55.596	0.314	0.280	0.366	0.238	0.422	55.200	0.726	29.603	23.827	26.715	19.856
DQ485256_CH	46.900	43.100	45.000	43.700	29.543	22.984	25.872	21.600	44.585	55.415	0.327	0.272	0.351	0.246	0.422	54.700	0.727	28.339	23.285	27.978	20.397
DQ485258_CH	46.800	43.300	45.050	43.700	29.603	22.984	25.812	21.600	44.585	55.415	0.325	0.274	0.354	0.241	0.421	54.600	0.727	28.520	23.466	27.798	20.217
DQ485261_CH	46.400	43.500	44.950	43.300	29.904	23.045	25.692	21.360	44.404	55.596	0.321	0.276	0.361	0.235	0.419	55.500	0.724	29.242	23.646	27.437	19.675
DQ485269_CH	46.600	43.100	44.850	44.600	29.362	22.744	25.872	22.022	44.765	55.235	0.328	0.273	0.340	0.259	0.431	56.400	0.722	27.437	23.285	27.978	21.300
DQ485271_CH	46.600	43.700	45.150	42.200	29.904	22.984	25.933	21.179	44.164	55.836	0.328	0.270	0.366	0.231	0.409	55.000	0.724	29.603	23.105	28.159	19.134
DQ486859_CH	47.500	42.800	45.150	42.100	30.144	23.045	25.752	21.059	44.103	55.897	0.324	0.278	0.378	0.218	0.404	54.200	0.726	30.325	23.646	27.617	18.412
E41_CH	48.000	42.800	45.400	42.400	29.844	23.045	25.752	21.360	44.404	55.596	0.326	0.277	0.373	0.223	0.408	54.200	0.726	29.783	23.646	27.798	18.773
EF579731_CH	47.100	43.500	45.300	42.200	29.663	22.623	26.053	21.661	44.284	55.716	0.339	0.263	0.360	0.236	0.406	54.700	0.720	28.881	22.383	28.881	19.856
EF579732_CH	46.600	43.000	44.800	44.200	29.783	22.984	25.632	21.600	44.585	55.415	0.327	0.278	0.351	0.245	0.425	55.600	0.723	28.159	23.466	27.617	20.758
EF579733_CH	46.900	43.100	45.000	42.600	29.783	22.684	25.993	21.540	44.224	55.776	0.335	0.267	0.360	0.238	0.411	55.400	0.721	28.881	22.744	28.520	19.856
EU583503_CH	46.900	43.900	45.400	43.700	29.603	23.165	25.572	21.661	44.826	55.174	0.321	0.276	0.358	0.240	0.422	55.800	0.725	28.881	23.646	27.437	20.036
FJ011441_CH	47.100	43.300	45.200	42.600	29.603	22.563	26.053	21.781	44.344	55.656	0.335	0.265	0.358	0.240	0.411	55.500	0.722	28.881	22.563	28.520	20.036
FJ011442_CH	47.100	43.300	45.200	42.200	29.663	22.443	26.113	21.781	44.224	55.776	0.337	0.261	0.360	0.240	0.407	55.600	0.721	29.061	22.202	28.700	20.036
FJ011443_CH	47.300	43.300	45.300	42.400	29.603	22.563	26.053	21.781	44.344	55.656	0.337	0.263	0.358	0.240	0.409	55.600	0.722	28.881	22.383	28.700	20.036
FJ011448_CH	47.100	43.700	45.400	42.800	29.603	22.744	25.872	21.781	44.525	55.475	0.333	0.267	0.358	0.240	0.413	55.800	0.721	28.881	22.744	28.339	20.036
FJ217665_CH	47.500	43.700	45.600	43.500	29.422	23.045	25.692	21.841	44.886	55.114	0.332	0.269	0.350	0.247	0.420	58.700	0.719	28.159	22.924	28.339	20.578
FJ217666t_CH	47.700	43.700	45.700	43.500	29.362	22.984	25.692	21.961	44.946	55.054	0.333	0.267	0.349	0.249	0.420	56.300	0.719	28.159	22.744	28.339	20.758
FJ426563_CH	46.900	43.300	45.100	42.600	29.603	22.563	26.113	21.721	44.284	55.716	0.337	0.265	0.357	0.239	0.410	55.700	0.721	28.700	22.563	28.700	20.036
FJ480789_CH	47.800	42.600	45.200	42.100	30.205	23.045	25.632	21.119	44.164	55.836	0.329	0.274	0.376	0.221	0.403	54.700	0.723	29.964	23.285	27.978	18.773
FJ480790_CH	47.300	43.500	45.400	42.600	29.543	22.623	25.993	21.841	44.465	55.535	0.335	0.265	0.358	0.240	0.411	55.600	0.722	28.881	22.563	28.520	20.036
FJ480791_CH	46.800	43.300	45.050	44.400	29.663	23.225	25.511	21.600	44.826	55.174	0.319	0.281	0.353	0.249	0.430	55.000	0.733	28.339	24.007	27.256	20.397
FJ480792_CH	47.300	43.700	45.500	42.600	29.483	22.563	25.993	21.961	44.525	55.475	0.335	0.265	0.357	0.239	0.411	55.800	0.722	28.881	22.563	28.520	20.036
FJ480796_CH	46.900	43.500	45.200	43.000	30.024	23.165	25.511	21.300	44.465	55.535	0.321	0.277	0.367	0.231	0.415	55.000	0.724	29.603	23.646	27.437	19.314
FJ480797_CH	46.800	43.500	45.150	43.000	30.024	23.165	25.572	21.239	44.404	55.596	0.321	0.276	0.367	0.231	0.415	55.200	0.723	29.603	23.646	27.437	19.314
FJ480798_CH	46.900	44.000	45.450	44.400	29.182	23.045	25.692	22.082	45.126	54.874	0.325	0.272	0.343	0.256	0.431	56.100	0.727	27.798	23.285	27.798	21.119
FJ480799_CH	47.300	43.300	45.300	42.600	29.483	22.503	26.113	21.901	44.404	55.596	0.334	0.262	0.358	0.242	0.411	55.700	0.724	28.881	22.383	28.520	20.217

FJ480800_CH	46.900	43.700	45.300	43.700	30.144	23.646	25.090	21.119	44.765	55.235	0.306	0.291	0.373	0.223	0.422	54.300	0.731	30.144	24.910	26.173	18.773
FJ480801_CH	46.600	43.300	44.950	43.300	29.904	22.984	25.692	21.420	44.404	55.596	0.319	0.276	0.365	0.236	0.419	55.200	0.723	29.422	23.646	27.256	19.675
FJ480802_CH	46.600	43.300	44.950	43.500	29.904	23.045	25.632	21.420	44.465	55.535	0.317	0.279	0.365	0.236	0.420	55.200	0.723	29.422	23.827	27.076	19.675
FJ480803_CH	46.900	43.700	45.300	43.000	30.205	23.466	25.271	21.059	44.525	55.475	0.312	0.285	0.375	0.220	0.415	54.400	0.728	30.325	24.368	26.715	18.592
FJ480804_CH	46.600	43.100	44.850	43.500	29.844	22.984	25.752	21.420	44.404	55.596	0.321	0.277	0.360	0.239	0.420	54.500	0.726	29.061	23.646	27.437	19.856
FJ480805_CH	46.900	43.700	45.300	43.100	30.144	23.466	25.271	21.119	44.585	55.415	0.312	0.285	0.373	0.223	0.417	54.400	0.730	30.144	24.368	26.715	18.773
FJ480806_CH	47.300	44.000	45.650	44.000	29.362	23.165	25.511	21.961	45.126	54.874	0.321	0.276	0.353	0.244	0.426	54.800	0.725	28.520	23.646	27.437	20.397
FJ480807_CH	46.900	43.700	45.300	43.100	30.205	23.526	25.211	21.059	44.585	55.415	0.310	0.287	0.375	0.220	0.417	54.700	0.729	30.325	24.549	26.534	18.592
FJ480809_CH	46.900	43.100	45.000	43.100	29.964	22.984	25.632	21.420	44.404	55.596	0.326	0.275	0.363	0.236	0.416	55.800	0.731	29.242	23.285	27.617	19.856
FJ480810_CH	47.300	43.300	45.300	42.800	29.964	23.045	25.572	21.420	44.465	55.535	0.322	0.275	0.369	0.231	0.413	55.600	0.727	29.783	23.466	27.437	19.314
FJ480812_CH	47.300	43.300	45.300	43.100	29.723	23.045	25.692	21.540	44.585	55.415	0.325	0.276	0.363	0.233	0.416	56.600	0.726	29.242	23.466	27.617	19.675
FJ480813_CH	46.900	43.300	45.100	42.400	29.603	22.503	26.173	21.721	44.224	55.776	0.335	0.265	0.360	0.238	0.409	55.900	0.719	29.061	22.563	28.520	19.856
FJ480818_CH	46.900	43.500	45.200	42.600	29.603	22.623	26.053	21.721	44.344	55.656	0.333	0.267	0.360	0.238	0.411	55.700	0.720	29.061	22.744	28.339	19.856
FJ480819_CH	47.100	43.500	45.300	42.800	29.603	22.684	25.933	21.781	44.465	55.535	0.333	0.267	0.358	0.240	0.413	55.600	0.723	28.881	22.744	28.339	20.036
FJ480820_CH	46.800	43.300	45.050	42.800	30.325	23.285	25.391	20.999	44.284	55.716	0.312	0.283	0.378	0.224	0.414	56.700	0.730	30.505	24.188	26.715	18.592
FJ608334_CH	47.300	43.700	45.500	44.000	29.543	23.165	25.451	21.841	45.006	54.994	0.321	0.277	0.354	0.245	0.426	56.800	0.721	28.520	23.646	27.437	20.397
FJ608335_CH	46.900	43.500	45.200	43.000	30.084	23.285	25.451	21.179	44.465	55.535	0.312	0.285	0.376	0.220	0.415	54.300	0.729	30.325	24.368	26.715	18.592
FJ608336_CH	47.500	43.700	45.600	43.300	29.723	23.165	25.451	21.661	44.826	55.174	0.327	0.274	0.358	0.240	0.419	56.600	0.717	28.881	23.285	27.798	20.036
FJ608338_CH	47.500	43.500	45.500	42.400	29.964	23.165	25.572	21.300	44.465	55.535	0.323	0.274	0.371	0.225	0.409	53.700	0.729	29.964	23.466	27.617	18.953
FJ608343_CH	47.500	43.900	45.700	43.300	29.603	23.165	25.511	21.721	44.886	55.114	0.324	0.275	0.359	0.237	0.419	56.700	0.720	29.061	23.466	27.617	19.856
FJ608345_CH	47.500	43.300	45.400	42.600	29.964	23.045	25.572	21.420	44.465	55.535	0.329	0.272	0.366	0.233	0.411	56.200	0.719	29.422	23.105	27.978	19.495
FJ608348_CH	46.900	43.500	45.200	43.000	30.084	23.225	25.451	21.239	44.465	55.535	0.314	0.283	0.374	0.223	0.415	54.700	0.729	30.144	24.188	26.895	18.773
FJ608349_CH	46.800	43.000	44.900	41.500	30.445	22.744	25.812	20.999	43.742	56.258	0.329	0.268	0.381	0.224	0.400	58.600	0.721	30.505	22.744	27.978	18.773
FJ608350_CH	47.300	43.500	45.400	43.100	29.904	23.285	25.451	21.360	44.645	55.355	0.312	0.285	0.373	0.222	0.417	54.400	0.729	30.144	24.368	26.715	18.773
FJ608351_CH	46.900	43.000	44.950	41.500	30.385	22.684	25.812	21.119	43.803	56.197	0.332	0.266	0.377	0.226	0.400	55.400	0.722	30.325	22.563	28.159	18.953
FJ754271_CH	46.600	43.300	44.950	42.100	30.024	22.804	25.993	21.179	43.983	56.017	0.329	0.268	0.368	0.231	0.407	54.900	0.720	29.783	22.924	28.159	19.134
FJ882014_CH	47.500	43.900	45.700	43.700	29.904	23.586	25.090	21.420	45.006	54.994	0.305	0.286	0.373	0.227	0.422	54.500	0.723	30.144	24.549	26.173	19.134
FJ882015_CH	46.900	43.500	45.200	43.100	29.964	23.225	25.511	21.300	44.525	55.475	0.314	0.283	0.371	0.225	0.417	54.500	0.730	29.964	24.188	26.895	18.953
GQ245780_CH	47.100	43.500	45.300	43.300	29.422	22.623	25.933	22.022	44.645	55.355	0.335	0.265	0.350	0.250	0.419	56.000	0.725	28.159	22.563	28.520	20.758
GQ245781_CH	46.800	43.900	45.350	42.400	29.603	22.563	26.053	21.781	44.344	55.656	0.335	0.263	0.361	0.240	0.409	54.900	0.722	29.061	22.383	28.520	20.036
GQ245782_CH	47.500	43.300	45.400	42.800	29.362	22.503	26.113	22.022	44.525	55.475	0.337	0.263	0.354	0.244	0.413	55.600	0.722	28.520	22.383	28.700	20.397
GQ245783_CH	47.300	43.300	45.300	42.800	29.603	22.623	25.933	21.841	44.465	55.535	0.331	0.267	0.360	0.240	0.413	55.500	0.721	29.061	22.744	28.159	20.036
GQ245784_CH	47.800	43.300	45.550	42.400	29.543	22.744	25.933	21.781	44.525	55.475	0.332	0.266	0.362	0.235	0.409	55.600	0.715	29.242	22.744	28.339	19.675
GQ245785_CH	47.300	43.500	45.400	43.000	29.422	22.563	25.993	22.022	44.585	55.415	0.335	0.267	0.358	0.238	0.412	55.700	0.722	28.700	22.563	28.339	20.397
GQ245786_CH	47.300	43.300	45.300	42.800	29.543	22.623	25.993	21.841	44.465	55.535	0.333	0.267	0.358	0.239	0.413	55.400	0.723	28.881	22.744	28.339	20.036
GQ245787_CH	46.900	43.500	45.200	42.800	29.603	22.623	25.993	21.781	44.404	55.596	0.333	0.267	0.358	0.240	0.413	55.400	0.723	28.881	22.744	28.339	20.036
GQ245788_CH	47.100	43.300	45.200	43.100	29.603	22.684	25.872	21.841	44.525	55.475	0.329	0.270	0.357	0.241	0.417	55.600	0.723	28.881	22.924	27.978	20.217
GQ245790_CH	46.900	43.300	45.100	43.700	29.723	22.984	25.632	21.661	44.645	55.355	0.325	0.274	0.357	0.246	0.422	55.900	0.720	28.700	23.285	27.617	20.397
GQ245791_CH	46.900	43.500	45.200	43.000	29.543	22.623	25.993	21.841	44.465	55.535	0.335	0.265	0.354	0.246	0.415	55.100	0.723	28.520	22.563	28.520	20.397
GQ245792_CH	47.100	43.700	45.400	43.100	29.483	22.744	25.872	21.901	44.645	55.355	0.331	0.269	0.356	0.241	0.417	55.600	0.718	28.700	22.924	28.159	20.217
GQ245796_CH	47.500	44.000	45.750	43.500	30.024	23.767	24.970	21.239	45.006	54.994	0.306	0.289	0.376	0.223	0.420	54.800	0.725	30.325	24.729	26.173	18.773
GQ245797_CH	47.300	43.700	45.500	43.900	29.964	23.526	25.090	21.420	44.946	55.054	0.309	0.288	0.369	0.229	0.424	57.300	0.727	29.783	24.549	26.354	19.314
GQ245798_CH	46.900	44.000	45.450	43.100	30.084	23.526	25.211	21.179	44.705	55.295	0.312	0.284	0.372	0.225	0.418	55.000	0.727	30.144	24.368	26.715	18.773
GQ245799_CH	46.900	43.700	45.300	43.300	30.205	23.586	25.150	21.059	44.645	55.355	0.308	0.289	0.376	0.221	0.419	55.300	0.721	30.325	24.729	26.354	18.592

GQ245802_CH	47.100	43.700	45.400	43.500	29.964	23.345	25.271	21.420	44.765	55.235	0.313	0.281	0.368	0.232	0.420	54.500	0.727	29.783	24.007	26.715	19.495
GQ245803_CH	46.900	43.500	45.200	42.800	30.024	23.165	25.572	21.239	44.404	55.596	0.317	0.281	0.374	0.223	0.413	54.500	0.729	30.144	24.007	27.076	18.773
GQ245804_CH	47.300	43.500	45.400	42.800	29.904	23.225	25.572	21.300	44.525	55.475	0.316	0.280	0.373	0.224	0.414	54.500	0.727	30.144	24.007	27.076	18.773
GQ245805_CH	47.100	43.900	45.500	43.100	29.844	23.345	25.451	21.360	44.705	55.295	0.321	0.279	0.366	0.228	0.416	54.600	0.728	29.422	23.827	27.437	19.314
GQ245806_CH	47.100	44.200	45.650	42.800	29.904	23.406	25.391	21.300	44.705	55.295	0.315	0.279	0.372	0.222	0.413	55.500	0.727	30.144	24.007	27.076	18.773
GQ245807_CH	46.900	43.700	45.300	43.100	30.144	23.345	25.271	21.239	44.585	55.415	0.312	0.284	0.374	0.225	0.418	54.300	0.729	30.144	24.368	26.715	18.773
GQ245808_CH	47.100	43.900	45.500	43.000	29.964	23.406	25.391	21.239	44.645	55.355	0.314	0.283	0.373	0.223	0.415	54.700	0.731	30.144	24.188	26.895	18.773
GQ245809_CH	46.900	43.700	45.300	44.000	29.964	23.526	25.150	21.360	44.886	55.114	0.307	0.290	0.369	0.230	0.426	57.200	0.730	29.783	24.729	26.173	19.314
GQ245810_CH	47.300	43.900	45.600	43.100	29.964	23.466	25.271	21.300	44.765	55.235	0.314	0.284	0.370	0.225	0.418	54.900	0.727	29.964	24.368	26.895	18.773
GQ245811_CH	47.100	43.700	45.400	43.900	29.964	23.526	25.150	21.360	44.886	55.114	0.309	0.288	0.368	0.230	0.424	57.300	0.728	29.783	24.549	26.354	19.314
GQ245812_CH	46.900	43.900	45.400	43.000	29.904	23.225	25.511	21.360	44.585	55.415	0.313	0.282	0.374	0.223	0.415	54.000	0.729	30.144	24.188	26.895	18.773
GQ245813_CH	47.300	43.700	45.500	43.700	29.904	23.466	25.211	21.420	44.886	55.114	0.310	0.285	0.368	0.232	0.423	57.300	0.726	29.783	24.368	26.534	19.314
GQ245814_CH	47.100	43.700	45.400	43.000	29.844	23.345	25.572	21.239	44.585	55.415	0.317	0.283	0.371	0.222	0.415	53.800	0.730	29.964	24.188	27.076	18.773
GQ245815_CH	47.300	43.900	45.600	42.800	29.904	23.345	25.451	21.300	44.645	55.355	0.324	0.277	0.368	0.225	0.412	55.100	0.724	29.603	23.646	27.617	19.134
GQ245816_CH	46.900	43.700	45.300	42.400	30.265	23.345	25.391	20.999	44.344	55.656	0.317	0.279	0.377	0.219	0.409	54.400	0.724	30.505	23.827	27.076	18.592
GQ245817_CH	47.700	43.700	45.700	43.300	30.084	23.646	25.030	21.239	44.886	55.114	0.311	0.286	0.374	0.223	0.417	55.800	0.730	30.144	24.368	26.534	18.953
GQ245819_CH	46.800	43.700	45.250	42.200	30.084	23.105	25.692	21.119	44.224	55.776	0.326	0.277	0.371	0.221	0.407	53.900	0.730	29.964	23.646	27.798	18.592
GU166154_CH	46.800	43.300	45.050	42.600	29.663	22.503	26.113	21.721	44.224	55.776	0.337	0.263	0.356	0.243	0.411	55.200	0.721	28.700	22.383	28.700	20.217
GZ19_CH	47.100	42.800	44.950	42.200	30.024	22.864	25.933	21.179	44.043	55.957	0.328	0.273	0.372	0.225	0.406	54.700	0.724	29.783	23.285	27.978	18.953
HD48_CH	47.300	43.000	45.150	42.100	30.084	22.984	25.812	21.119	44.103	55.897	0.331	0.275	0.373	0.222	0.404	53.700	0.725	29.783	23.466	28.159	18.592
HM748945_CH	46.600	43.000	44.800	42.800	29.783	22.383	26.113	21.721	44.103	55.897	0.337	0.262	0.358	0.244	0.411	55.800	0.722	28.700	22.202	28.520	20.578
HM748946_CH	47.100	43.100	45.100	42.600	29.783	22.563	25.933	21.721	44.284	55.716	0.336	0.262	0.359	0.243	0.410	58.500	0.723	28.881	22.202	28.520	20.397
HM748947_CH	47.100	43.500	45.300	42.800	29.964	23.225	25.572	21.239	44.465	55.535	0.327	0.276	0.366	0.226	0.411	55.100	0.728	29.422	23.466	27.798	19.314
JF343539_CH	46.600	43.700	45.150	43.700	29.844	23.105	25.511	21.540	44.645	55.355	0.317	0.279	0.362	0.238	0.422	55.700	0.725	29.242	23.827	27.076	19.856
JN400897_CH	46.800	43.700	45.250	43.300	29.964	23.285	25.451	21.300	44.585	55.415	0.317	0.283	0.368	0.229	0.419	54.500	0.731	29.603	24.188	27.076	19.134
JQ013855_CH	46.900	43.500	45.200	43.500	29.964	23.406	25.391	21.239	44.645	55.355	0.312	0.286	0.368	0.228	0.421	55.400	0.731	29.783	24.549	26.715	18.953
JQ013856_CH	47.500	43.900	45.700	43.300	29.964	23.586	25.150	21.300	44.886	55.114	0.309	0.286	0.373	0.225	0.420	55.200	0.723	30.144	24.549	26.534	18.773
JQ013857_CH	47.100	43.500	45.300	43.000	29.964	23.165	25.511	21.360	44.525	55.475	0.317	0.279	0.372	0.226	0.414	55.200	0.720	29.964	23.827	27.076	19.134
JQ013858_CH	47.100	43.300	45.200	43.100	29.964	23.225	25.511	21.300	44.525	55.475	0.317	0.281	0.369	0.228	0.417	55.500	0.720	29.783	24.007	27.076	19.134
JQ013859_CH	46.900	43.500	45.200	43.700	29.964	23.466	25.331	21.239	44.705	55.295	0.310	0.288	0.368	0.228	0.423	55.400	0.732	29.783	24.729	26.534	18.953
JQ013860_CH	46.900	43.900	45.400	43.300	30.024	23.466	25.271	21.239	44.705	55.295	0.310	0.286	0.374	0.223	0.419	54.700	0.727	30.144	24.549	26.534	18.773
JQ013861_CH	46.900	42.800	44.850	42.100	30.205	22.684	25.872	21.239	43.923	56.077	0.331	0.268	0.369	0.233	0.407	55.300	0.724	29.783	22.744	28.159	19.314
JQ013863_CH	46.900	43.700	45.300	43.100	29.663	23.105	25.752	21.480	44.585	55.415	0.322	0.276	0.361	0.235	0.418	55.900	0.725	29.242	23.646	27.617	19.495
JQ013864_CH	47.100	43.700	45.400	41.900	29.723	22.503	26.053	21.721	44.224	55.776	0.336	0.260	0.364	0.234	0.404	55.400	0.720	29.422	22.202	28.700	19.675
JQ013865_CH	47.300	43.900	45.600	43.500	29.904	23.526	25.211	21.360	44.886	55.114	0.307	0.286	0.373	0.227	0.421	55.100	0.723	30.144	24.549	26.354	18.953
JQ013866_CH	47.100	43.500	45.300	43.900	29.904	23.526	25.271	21.300	44.826	55.174	0.310	0.288	0.365	0.231	0.425	55.400	0.731	29.603	24.729	26.534	19.134
JQ013867_CH	46.900	43.500	45.200	44.000	29.844	23.466	25.331	21.360	44.826	55.174	0.310	0.289	0.363	0.230	0.426	55.600	0.730	29.422	24.729	26.534	19.314
JQ013868_CH	47.300	44.000	45.650	43.900	29.964	23.767	24.970	21.300	45.066	54.934	0.303	0.290	0.372	0.227	0.425	54.500	0.727	30.144	24.910	25.993	18.953
JQ013869_CH	47.100	43.300	45.200	43.900	29.844	23.345	25.391	21.420	44.765	55.235	0.312	0.285	0.365	0.233	0.424	55.900	0.720	29.422	24.368	26.715	19.495
JQ013870_CH	46.900	44.000	45.450	43.900	29.964	23.586	25.090	21.360	44.946	55.054	0.307	0.286	0.368	0.230	0.424	54.800	0.731	29.783	24.549	26.354	19.314
JQ013871_CH	46.900	44.000	45.450	44.000	29.964	23.646	25.030	21.360	45.006	54.994	0.305	0.288	0.368	0.230	0.426	54.700	0.731	29.783	24.729	26.173	19.314
JQ013873_CH	47.300	43.900	45.600	43.500	30.024	23.586	25.090	21.300	44.886	55.114	0.307	0.286	0.374	0.225	0.420	54.600	0.729	30.144	24.549	26.354	18.953
JQ013874_CH	47.300	43.700	45.500	43.900	29.844	23.526	25.211	21.420	44.946	55.054	0.307	0.288	0.368	0.230	0.425	55.800	0.727	29.783	24.729	26.354	19.134
JQ013876_CH	47.100	43.500	45.300	43.300	29.783	23.225	25.572	21.420	44.645	55.355	0.318	0.280	0.363	0.233	0.420	55.500	0.728	29.422	24.007	27.256	19.314

JQ013877_CH	47.300	44.000	45.650	43.700	29.964	23.586	25.030	21.420	45.006	54.994	0.308	0.287	0.372	0.226	0.421	55.000	0.728	29.964	24.549	26.354	19.134
JQ013878_CH	47.300	43.700	45.500	43.100	30.084	23.466	25.211	21.239	44.705	55.295	0.310	0.284	0.376	0.223	0.417	55.200	0.722	30.325	24.368	26.534	18.773
JQ015295_CH	47.300	44.000	45.650	43.500	30.024	23.646	25.030	21.300	44.946	55.054	0.307	0.286	0.374	0.225	0.420	55.000	0.723	30.144	24.549	26.354	18.953
JQ015296_CH	47.300	43.900	45.600	43.500	30.024	23.586	25.090	21.300	44.886	55.114	0.307	0.286	0.374	0.226	0.420	55.000	0.722	30.144	24.549	26.354	18.953
JQ015297_CH	47.100	44.000	45.550	43.000	30.024	23.406	25.271	21.300	44.705	55.295	0.314	0.280	0.374	0.225	0.415	55.500	0.719	30.144	24.007	26.895	18.953
JQ894778_CH	47.500	44.000	45.750	41.500	29.663	22.563	25.993	21.781	44.344	55.656	0.338	0.261	0.369	0.225	0.398	55.500	0.723	29.783	22.202	28.700	19.314
JX193075_CH	46.800	43.300	45.050	43.700	29.543	22.864	25.872	21.721	44.585	55.415	0.325	0.274	0.355	0.239	0.420	54.400	0.723	28.520	23.466	27.798	20.217
JX193076_CH	46.900	44.000	45.450	41.900	29.844	22.864	25.872	21.420	44.284	55.716	0.333	0.265	0.365	0.230	0.405	55.200	0.727	29.603	22.744	28.520	19.134
JX519467_CH	47.100	43.900	45.500	41.500	29.904	22.623	25.933	21.540	44.164	55.836	0.333	0.265	0.374	0.222	0.399	55.300	0.720	30.144	22.563	28.339	18.953
JX840452_CH	47.300	44.000	45.650	43.700	29.964	23.586	25.030	21.420	45.006	54.994	0.307	0.284	0.371	0.230	0.422	54.900	0.726	29.964	24.368	26.354	19.314
JX840453_CH	47.300	44.200	45.750	43.700	29.904	23.586	25.030	21.480	45.066	54.934	0.307	0.284	0.372	0.231	0.422	55.000	0.728	29.964	24.368	26.354	19.314
JX840454_CH	47.300	44.000	45.650	43.700	29.964	23.646	25.030	21.360	45.006	54.994	0.307	0.286	0.371	0.228	0.422	55.300	0.722	29.964	24.549	26.354	19.134
JX840455_CH	46.600	43.500	45.050	44.000	30.024	23.466	25.271	21.239	44.705	55.295	0.303	0.290	0.371	0.231	0.427	54.800	0.725	29.964	24.910	25.993	19.134
KC020114_CH	47.300	44.000	45.650	43.500	29.964	23.586	25.090	21.360	44.946	55.054	0.310	0.284	0.371	0.228	0.420	55.200	0.723	29.964	24.368	26.534	19.134
KC292514_CH	46.900	42.800	44.850	41.900	30.265	22.623	25.872	21.239	43.863	56.137	0.330	0.268	0.376	0.227	0.403	55.800	0.723	30.144	22.744	27.978	19.134
KC292517_CH	46.900	43.300	45.100	41.700	30.205	22.744	25.812	21.239	43.983	56.017	0.332	0.266	0.374	0.226	0.401	55.900	0.723	30.144	22.563	28.159	19.134
KC292519_CH	47.500	43.100	45.300	43.100	29.964	23.165	25.451	21.420	44.585	55.415	0.315	0.279	0.372	0.228	0.416	55.200	0.717	29.964	23.827	26.895	19.314
KC461214_CH	47.300	44.000	45.650	41.700	29.663	22.623	25.993	21.721	44.344	55.656	0.337	0.261	0.364	0.232	0.403	55.100	0.714	29.422	22.383	28.881	19.314
KC489471_CH	47.100	42.800	44.950	43.500	29.964	23.285	25.572	21.179	44.465	55.535	0.314	0.283	0.369	0.232	0.420	55.400	0.720	29.603	24.188	26.895	19.314
KC542892_CH	47.100	43.700	45.400	43.000	29.964	23.285	25.451	21.300	44.585	55.415	0.317	0.281	0.371	0.225	0.415	54.800	0.727	29.964	24.007	27.076	18.953
KC542893t_CH	47.100	43.700	45.400	42.600	29.964	23.165	25.572	21.300	44.465	55.535	0.321	0.276	0.371	0.225	0.411	54.400	0.727	29.964	23.646	27.437	18.953
KC542895_CH	47.500	43.500	45.500	43.100	29.783	23.105	25.511	21.600	44.705	55.295	0.327	0.274	0.360	0.237	0.417	56.500	0.719	29.061	23.285	27.798	19.856
KC542897_CH	47.500	43.100	45.300	43.000	29.603	22.804	25.872	21.721	44.525	55.475	0.336	0.266	0.354	0.245	0.415	56.300	0.724	28.520	22.563	28.520	20.397
KC542898_CH	47.300	43.100	45.200	42.800	29.904	22.924	25.692	21.480	44.404	55.596	0.327	0.272	0.365	0.236	0.413	55.900	0.726	29.422	23.105	27.798	19.675
KC542899_CH	46.900	43.700	45.300	43.300	29.964	23.406	25.391	21.239	44.645	55.355	0.312	0.287	0.371	0.223	0.419	54.500	0.729	29.964	24.549	26.715	18.773
KC542902_CH	46.800	43.300	45.050	43.900	29.723	23.045	25.632	21.600	44.645	55.355	0.323	0.274	0.354	0.250	0.425	55.000	0.727	28.520	23.466	27.617	20.397
KC542903_CH	47.300	43.500	45.400	43.500	29.904	23.406	25.331	21.360	44.765	55.235	0.308	0.289	0.373	0.222	0.420	54.400	0.730	30.144	24.729	26.354	18.773
KC542905_CH	47.100	43.900	45.500	43.700	30.024	23.586	25.090	21.300	44.886	55.114	0.305	0.288	0.374	0.225	0.422	54.700	0.723	30.144	24.729	26.173	18.953
KC542906_CH	46.900	43.700	45.300	43.500	30.024	23.466	25.271	21.239	44.705	55.295	0.309	0.286	0.371	0.228	0.421	55.000	0.720	29.964	24.549	26.534	18.953
KC542907_CH	46.900	43.300	45.100	43.900	30.024	23.466	25.271	21.239	44.705	55.295	0.305	0.291	0.372	0.226	0.424	54.900	0.723	29.964	24.910	26.173	18.953
KC542908_CH	46.000	43.700	44.850	42.400	30.084	22.924	25.872	21.119	44.043	55.957	0.323	0.276	0.372	0.224	0.409	54.500	0.732	29.964	23.646	27.617	18.773
KC542909_CH	47.100	43.500	45.300	42.400	30.084	23.105	25.572	21.239	44.344	55.656	0.318	0.276	0.375	0.226	0.410	54.100	0.726	30.325	23.646	27.256	18.773
KC542910_CH	46.800	43.300	45.050	44.200	29.964	23.466	25.271	21.300	44.765	55.235	0.303	0.293	0.370	0.229	0.428	58.000	0.723	29.783	25.090	25.993	19.134
KC542911_CH	46.600	43.100	44.850	43.900	30.024	23.225	25.451	21.300	44.525	55.475	0.310	0.287	0.368	0.232	0.424	55.200	0.725	29.603	24.549	26.534	19.314
KC542912_CH	46.900	44.000	45.450	43.500	30.024	23.526	25.150	21.300	44.826	55.174	0.310	0.284	0.371	0.228	0.420	55.000	0.726	29.964	24.368	26.534	19.134
KF055273_CH	47.300	44.000	45.650	43.900	29.904	23.646	25.030	21.420	45.066	54.934	0.305	0.288	0.371	0.227	0.424	55.100	0.727	29.964	24.729	26.173	19.134
KF208469_CH	47.300	43.900	45.600	43.100	29.783	23.225	25.451	21.540	44.765	55.235	0.321	0.277	0.365	0.232	0.417	55.700	0.721	29.422	23.646	27.437	19.495
KJ136258_CH	46.800	43.300	45.050	43.700	30.084	23.406	25.331	21.179	44.585	55.415	0.310	0.286	0.370	0.229	0.422	55.400	0.719	29.783	24.549	26.534	19.134
KJ184574_CH	46.900	44.000	45.450	43.900	29.783	23.767	25.271	21.179	44.946	55.054	0.309	0.290	0.365	0.229	0.425	56.200	0.728	29.783	24.729	26.354	19.134
KJ184575_CH	46.600	44.200	45.400	43.300	30.024	23.586	25.271	21.119	44.705	55.295	0.307	0.288	0.375	0.222	0.420	54.600	0.726	30.325	24.729	26.354	18.592
KJ184576_CH	47.800	44.200	46.000	43.500	29.783	23.646	25.030	21.540	45.187	54.813	0.312	0.284	0.370	0.228	0.420	55.400	0.722	29.783	24.368	26.715	19.134
KJ184577_CH	47.100	43.300	45.200	43.500	29.904	23.285	25.451	21.360	44.645	55.355	0.310	0.285	0.372	0.226	0.419	55.500	0.721	29.964	24.368	26.534	19.134
KJ184578_CH	47.300	44.000	45.650	43.100	30.084	23.586	25.090	21.239	44.826	55.174	0.310	0.284	0.376	0.223	0.417	55.000	0.721	30.325	24.368	26.534	18.773
KJ184581_CH	47.300	44.000	45.650	44.000	29.964	23.767	24.910	21.360	45.126	54.874	0.303	0.291	0.371	0.228	0.426	55.100	0.723	29.964	24.910	25.993	19.134

KJ184586_CH	47.500	44.200	45.850	44.000	29.844	23.947	24.910	21.300	45.247	54.753	0.304	0.293	0.370	0.224	0.426	55.300	0.723	29.964	25.090	25.993	18.953
KJ184587_CH	46.900	43.300	45.100	43.700	30.024	23.345	25.331	21.300	44.645	55.355	0.307	0.286	0.372	0.229	0.422	54.900	0.720	29.964	24.549	26.354	19.134
KJ184588t_CH	47.300	44.000	45.650	44.200	29.964	23.767	24.850	21.420	45.187	54.813	0.301	0.291	0.371	0.230	0.428	55.100	0.724	29.964	24.910	25.812	19.314
KJ184589_CH	46.900	44.000	45.450	43.900	30.024	23.646	25.030	21.300	44.946	55.054	0.305	0.288	0.371	0.228	0.424	54.800	0.728	29.964	24.729	26.173	19.134
KJ184591_CH	46.200	43.700	44.950	42.400	30.084	22.984	25.812	21.119	44.103	55.897	0.323	0.276	0.372	0.224	0.409	54.200	0.732	29.964	23.646	27.617	18.773
KJ184592_CH	46.900	43.300	45.100	43.700	30.024	23.406	25.331	21.239	44.645	55.355	0.307	0.288	0.372	0.226	0.422	54.800	0.721	29.964	24.729	26.354	18.953
KJ184593_CH	46.800	43.300	45.050	43.900	30.024	23.406	25.331	21.239	44.645	55.355	0.305	0.291	0.373	0.227	0.424	55.000	0.725	29.964	24.910	26.173	18.953
KJ184594_CH	47.300	43.900	45.600	43.700	29.964	23.586	25.090	21.360	44.946	55.054	0.307	0.286	0.371	0.228	0.422	55.200	0.721	29.964	24.549	26.354	19.134
KJ184595_CH	46.900	44.000	45.450	43.100	30.024	23.406	25.271	21.300	44.705	55.295	0.312	0.282	0.374	0.225	0.417	54.600	0.725	30.144	24.188	26.715	18.953
KJ184596_CH	46.900	44.000	45.450	43.000	30.084	23.406	25.271	21.239	44.645	55.355	0.312	0.282	0.376	0.223	0.415	54.500	0.726	30.325	24.188	26.715	18.773
KJ184597_CH	46.900	43.100	45.000	44.200	30.024	23.526	25.211	21.239	44.765	55.235	0.306	0.291	0.368	0.231	0.428	55.000	0.724	29.603	24.910	26.173	19.314
KJ184600_CH	47.300	43.900	45.600	44.000	29.723	23.586	25.211	21.480	45.066	54.934	0.307	0.288	0.364	0.232	0.427	56.500	0.726	29.603	24.729	26.354	19.314
KJ450977_CH	47.100	43.700	45.400	43.300	29.783	23.225	25.511	21.480	44.705	55.295	0.314	0.282	0.369	0.227	0.419	54.900	0.726	29.783	24.188	26.895	19.134
KJ450978_CH	46.900	43.500	45.200	43.700	30.024	23.406	25.271	21.300	44.705	55.295	0.310	0.286	0.370	0.229	0.422	55.400	0.714	29.783	24.549	26.534	19.134
KJ450979_CH	47.100	43.000	45.050	42.100	30.144	22.684	25.812	21.360	44.043	55.957	0.331	0.265	0.369	0.236	0.407	55.400	0.725	29.783	22.563	28.159	19.495
KJ450980_CH	47.300	44.000	45.650	43.500	30.084	23.646	24.970	21.300	44.946	55.054	0.305	0.286	0.376	0.225	0.420	55.200	0.720	30.325	24.549	26.173	18.953
KJ525670_CH	47.100	43.900	45.500	43.300	30.084	23.586	25.150	21.179	44.765	55.235	0.310	0.286	0.374	0.223	0.419	55.000	0.724	30.144	24.549	26.534	18.773
KJ525683_CH	46.800	43.500	45.150	42.100	30.205	22.984	25.692	21.119	44.103	55.897	0.320	0.274	0.377	0.223	0.407	54.200	0.726	30.505	23.466	27.437	18.592
KJ525684_CH	46.900	43.500	45.200	42.100	30.084	22.924	25.752	21.239	44.164	55.836	0.323	0.272	0.376	0.223	0.406	54.100	0.727	30.325	23.285	27.617	18.773
KJ525686_CH	47.100	43.700	45.400	43.300	30.205	23.586	25.090	21.119	44.705	55.295	0.309	0.285	0.376	0.225	0.419	55.800	0.726	30.325	24.368	26.354	18.953
KJ525687_CH	46.800	43.100	44.950	43.500	30.024	23.285	25.511	21.179	44.465	55.535	0.314	0.287	0.368	0.226	0.420	54.800	0.731	29.603	24.549	26.895	18.953
KJ525688_CH	47.300	43.900	45.600	44.000	29.904	23.706	25.030	21.360	45.066	54.934	0.305	0.291	0.369	0.228	0.426	55.400	0.723	29.783	24.910	26.173	19.134
KJ525689_CH	46.800	43.100	44.950	43.900	30.144	23.466	25.271	21.119	44.585	55.415	0.306	0.291	0.372	0.226	0.424	55.400	0.717	29.964	24.910	26.173	18.953
KJ525690_CH	47.500	43.500	45.500	43.100	29.904	23.165	25.391	21.540	44.705	55.295	0.325	0.276	0.362	0.235	0.417	56.600	0.721	29.242	23.466	27.617	19.675
KJ525691_CH	47.500	44.000	45.750	43.500	29.904	23.586	25.090	21.420	45.006	54.994	0.310	0.284	0.371	0.228	0.420	55.000	0.724	29.964	24.368	26.534	19.134
KJ525693_CH	46.900	43.300	45.100	44.200	29.844	23.345	25.331	21.480	44.826	55.174	0.307	0.288	0.365	0.235	0.429	55.100	0.722	29.422	24.729	26.354	19.495
KJ525694_CH	47.300	44.000	45.650	43.500	29.964	23.586	25.090	21.360	44.946	55.054	0.310	0.284	0.371	0.228	0.420	55.000	0.723	29.964	24.368	26.534	19.134
KJ525695_CH	47.300	44.000	45.650	43.700	29.904	23.586	25.090	21.420	45.006	54.994	0.310	0.284	0.369	0.230	0.422	54.900	0.724	29.783	24.368	26.534	19.314
KJ525696_CH	46.900	43.700	45.300	43.700	30.084	23.586	25.150	21.179	44.765	55.235	0.305	0.290	0.374	0.226	0.423	54.700	0.719	30.144	24.910	26.173	18.773
KJ525697_CH	47.500	43.300	45.400	43.500	29.663	23.285	25.572	21.480	44.765	55.235	0.321	0.279	0.361	0.233	0.419	55.000	0.727	29.061	23.827	27.437	19.675
KJ525698_CH	47.100	43.700	45.400	43.000	29.783	23.225	25.632	21.360	44.585	55.415	0.327	0.276	0.364	0.226	0.412	55.200	0.727	29.242	23.466	27.798	19.495
KJ525699_CH	47.100	43.900	45.500	43.300	30.024	23.586	25.211	21.179	44.765	55.235	0.312	0.286	0.371	0.223	0.419	55.500	0.723	29.964	24.549	26.715	18.773
KJ525702_CH	46.900	43.100	45.000	44.200	29.603	23.165	25.632	21.600	44.765	55.235	0.325	0.280	0.351	0.245	0.427	55.600	0.722	28.159	23.827	27.617	20.397
KJ525704_CH	47.300	43.900	45.600	43.300	30.024	23.586	25.150	21.239	44.826	55.174	0.312	0.284	0.371	0.226	0.419	55.000	0.722	29.964	24.368	26.715	18.953
KJ525705_CH	47.100	44.200	45.650	43.500	30.024	23.646	25.030	21.300	44.946	55.054	0.307	0.286	0.373	0.225	0.420	54.600	0.726	30.144	24.549	26.354	18.953
KJ525706_CH	46.800	43.300	45.050	42.800	29.663	22.563	26.053	21.721	44.284	55.716	0.335	0.263	0.356	0.245	0.413	55.800	0.723	28.700	22.383	28.520	20.397
KJ525707_CH	47.500	43.900	45.700	43.500	30.084	23.646	24.970	21.300	44.946	55.054	0.307	0.284	0.374	0.228	0.420	57.000	0.723	30.144	24.368	26.354	19.134
KJ525708_CH	47.100	43.900	45.500	43.500	29.964	23.466	25.211	21.360	44.826	55.174	0.312	0.283	0.370	0.228	0.419	55.200	0.723	29.783	24.188	26.715	19.314
KJ525709_CH	46.600	43.500	45.050	42.600	30.024	23.045	25.752	21.179	44.224	55.776	0.321	0.279	0.372	0.224	0.411	54.200	0.733	29.964	23.827	27.437	18.773
KJ525710_CH	47.100	43.000	45.050	42.200	30.205	23.105	25.692	20.999	44.103	55.897	0.324	0.278	0.375	0.220	0.406	54.100	0.726	30.144	23.646	27.617	18.592
KJ525712_CH	47.300	44.000	45.650	43.700	29.844	23.586	25.150	21.420	45.006	54.994	0.309	0.284	0.369	0.230	0.422	55.300	0.722	29.783	24.368	26.534	19.314
KJ525714_CH	47.100	43.900	45.500	43.500	30.024	23.586	25.150	21.239	44.826	55.174	0.310	0.285	0.371	0.228	0.420	55.700	0.721	29.964	24.368	26.534	19.134
KJ525715_CH	47.300	43.700	45.500	43.700	29.964	23.526	25.150	21.360	44.886	55.114	0.310	0.285	0.369	0.230	0.422	55.600	0.721	29.783	24.368	26.534	19.314
KJ525716_CH	46.800	43.500	45.150	43.300	30.024	23.165	25.451	21.360	44.525	55.475	0.314	0.281	0.368	0.231	0.419	55.100	0.728	29.783	24.007	26.895	19.314

KJ525717_CH	46.900	43.000	44.950	43.000	29.904	22.744	25.812	21.540	44.284	55.716	0.331	0.271	0.359	0.240	0.415	56.000	0.726	29.061	22.924	27.978	20.036
KJ525718t_CH	46.400	43.300	44.850	42.800	29.964	23.045	25.872	21.119	44.164	55.836	0.323	0.281	0.368	0.224	0.413	54.400	0.734	29.603	24.007	27.617	18.773
KJ567597_CH	47.700	43.700	45.700	44.400	29.904	23.767	24.850	21.480	45.247	54.753	0.298	0.293	0.375	0.229	0.429	57.100	0.718	30.144	25.090	25.451	19.314
KJ600785_CH	47.300	43.500	45.400	42.600	29.603	22.563	25.933	21.901	44.465	55.535	0.335	0.261	0.358	0.244	0.411	57.800	0.723	28.881	22.202	28.520	20.397
KJ825877_CH	47.100	43.700	45.400	41.300	30.205	23.045	25.752	20.999	44.043	55.957	0.333	0.266	0.372	0.223	0.399	54.700	0.723	30.144	22.744	28.520	18.592
KJ825878_CH	46.600	43.300	44.950	44.200	29.904	23.406	25.391	21.300	44.705	55.295	0.308	0.289	0.366	0.232	0.427	55.800	0.723	29.422	24.729	26.354	19.495
KP064014_CH	46.900	43.900	45.400	43.100	30.144	23.526	25.211	21.119	44.645	55.355	0.312	0.283	0.374	0.226	0.417	55.200	0.723	30.144	24.188	26.715	18.953
KP064015_CH	47.100	43.900	45.500	43.100	30.084	23.466	25.211	21.239	44.705	55.295	0.312	0.281	0.374	0.228	0.417	55.700	0.720	30.144	24.007	26.715	19.134
KP064016_CH	46.800	44.000	45.400	43.500	30.024	23.466	25.211	21.300	44.765	55.235	0.307	0.286	0.374	0.225	0.420	54.000	0.728	30.144	24.549	26.354	18.953
KP064017_CH	46.600	44.200	45.400	43.000	30.084	23.526	25.331	21.059	44.585	55.415	0.308	0.285	0.376	0.222	0.417	54.100	0.725	30.505	24.549	26.534	18.412
KP064018_CH	46.000	43.000	44.500	43.000	30.084	22.684	25.933	21.300	43.983	56.017	0.319	0.277	0.370	0.230	0.414	55.400	0.722	29.783	23.646	27.256	19.314
KP064019_CH	46.800	43.300	45.050	43.100	30.205	23.345	25.391	21.059	44.404	55.596	0.312	0.284	0.374	0.224	0.417	54.700	0.718	30.144	24.368	26.715	18.773
KP064020_CH	46.900	44.200	45.550	43.300	30.084	23.586	25.090	21.239	44.826	55.174	0.310	0.284	0.373	0.225	0.419	54.600	0.727	30.144	24.368	26.534	18.953
KT381593_CH	47.500	42.600	45.050	42.200	30.205	23.045	25.692	21.059	44.103	55.897	0.322	0.280	0.379	0.218	0.406	54.400	0.726	30.325	23.827	27.437	18.412
KT760568_CH	46.200	43.700	44.950	43.700	29.422	22.864	26.053	21.661	44.525	55.475	0.329	0.266	0.348	0.255	0.423	57.300	0.737	27.978	22.924	28.339	20.758
KU140419_CH	47.300	43.300	45.300	42.600	29.603	22.623	25.993	21.781	44.404	55.596	0.336	0.265	0.358	0.240	0.411	56.100	0.722	28.881	22.563	28.520	20.036
KU175230_CH	46.200	43.300	44.750	43.500	29.483	22.684	26.173	21.661	44.344	55.656	0.333	0.263	0.348	0.258	0.421	55.400	0.736	27.978	22.563	28.520	20.939
KU175231_CH	46.200	43.700	44.950	43.700	29.422	22.864	26.053	21.661	44.525	55.475	0.329	0.266	0.348	0.255	0.423	57.300	0.737	27.978	22.924	28.339	20.758
KU175232_CH	46.000	43.300	44.650	43.500	29.663	22.744	26.053	21.540	44.284	55.716	0.331	0.265	0.351	0.255	0.421	55.600	0.737	28.159	22.744	28.339	20.758
KU200243_CH	47.500	43.300	45.400	44.800	29.302	23.285	25.511	21.901	45.187	54.813	0.318	0.280	0.349	0.251	0.432	56.100	0.731	27.978	24.007	27.256	20.758
KU200244_CH	47.500	43.500	45.500	43.900	29.543	23.285	25.511	21.661	44.946	55.054	0.321	0.279	0.357	0.241	0.424	56.500	0.732	28.700	23.827	27.437	20.036
KU200246_CH	47.700	43.900	45.800	43.700	29.302	23.406	25.632	21.661	45.066	54.934	0.321	0.275	0.355	0.243	0.423	54.500	0.730	28.700	23.646	27.617	20.036
KU200251_CH	48.000	43.500	45.750	43.700	29.362	23.105	25.572	21.961	45.066	54.934	0.321	0.275	0.359	0.242	0.422	56.400	0.715	28.881	23.466	27.437	20.217
KU200253_CH	47.100	43.500	45.300	43.500	30.024	23.526	25.271	21.179	44.705	55.295	0.309	0.288	0.371	0.226	0.421	55.500	0.718	29.964	24.729	26.534	18.773
KX765879_CH	46.000	43.100	44.550	43.700	29.543	22.684	26.173	21.600	44.284	55.716	0.331	0.265	0.348	0.258	0.423	54.800	0.739	27.978	22.744	28.339	20.939
MH105247_CH	46.200	43.300	44.750	43.300	29.422	22.623	26.294	21.661	44.284	55.716	0.336	0.263	0.343	0.256	0.421	57.100	0.735	27.798	22.563	28.881	20.758
MH105251_CH	46.900	44.000	45.450	43.300	30.265	23.706	24.970	21.059	44.765	55.235	0.303	0.291	0.380	0.218	0.419	54.300	0.721	30.686	24.910	25.993	18.412
MK006001_CH	46.900	43.700	45.300	41.700	29.422	22.142	26.474	21.961	44.103	55.897	0.347	0.246	0.353	0.250	0.403	55.200	0.724	28.520	21.119	29.783	20.578
MK342603_CH	47.100	43.100	45.100	42.800	29.904	22.383	25.752	21.961	44.344	55.656	0.328	0.271	0.368	0.235	0.411	55.800	0.721	29.242	23.105	27.978	19.675
MK611802_CH	47.300	43.100	45.200	42.600	29.723	22.383	25.933	21.961	44.344	55.656	0.332	0.269	0.367	0.236	0.409	55.700	0.721	29.061	22.924	28.339	19.675
MK764388h_CH	47.300	43.500	45.400	43.100	29.904	23.285	25.451	21.360	44.645	55.355	0.312	0.285	0.373	0.222	0.417	54.400	0.729	30.144	24.368	26.715	18.773
MK764389_CH	46.800	43.500	45.150	43.300	29.964	23.225	25.511	21.300	44.525	55.475	0.312	0.285	0.372	0.226	0.419	54.700	0.731	29.964	24.368	26.715	18.953
MK764390_CH	47.100	43.500	45.300	43.500	30.325	23.526	24.970	21.179	44.705	55.295	0.303	0.286	0.379	0.227	0.420	55.900	0.717	30.505	24.549	25.993	18.953
QY83_CH	47.100	43.500	45.300	41.700	29.663	22.443	26.233	21.661	44.103	55.897	0.343	0.258	0.359	0.237	0.403	55.200	0.723	29.061	22.022	29.242	19.675
SD22_CH	47.300	43.000	45.150	41.700	30.265	23.045	25.752	20.939	43.983	56.017	0.326	0.275	0.381	0.215	0.401	53.800	0.727	30.505	23.466	27.798	18.231
U62620_CH	46.600	42.600	44.600	43.100	29.783	22.864	26.113	21.239	44.103	55.897	0.326	0.278	0.363	0.234	0.417	56.400	0.731	29.061	23.646	27.798	19.495
ZH46_CH	47.100	42.800	44.950	41.200	30.205	22.684	26.113	20.999	43.682	56.318	0.340	0.265	0.375	0.218	0.394	53.200	0.719	29.964	22.563	28.881	18.592
FJ754272_DU	47.100	43.700	45.400	43.300	29.483	22.744	25.812	21.961	44.705	55.295	0.331	0.267	0.354	0.245	0.417	55.200	0.660	28.520	22.744	28.159	20.578
GD02_DU	48.000	42.400	45.200	42.100	30.084	22.984	25.752	21.179	44.164	55.836	0.327	0.276	0.376	0.220	0.404	54.300	0.659	30.144	23.466	27.798	18.592
GD04_DU	47.500	42.600	45.050	41.900	30.144	22.924	25.872	21.059	43.983	56.017	0.326	0.275	0.379	0.218	0.403	54.400	0.660	30.325	23.466	27.798	18.412
HM188393_DU	46.900	43.700	45.300	43.300	29.422	22.744	25.933	21.901	44.645	55.355	0.332	0.266	0.351	0.246	0.417	55.000	0.660	28.339	22.744	28.339	20.578
HM188396_DU	47.300	43.900	45.600	44.400	29.302	23.406	25.511	21.781	45.187	54.813	0.323	0.281	0.348	0.247	0.430	55.200	0.665	27.978	24.007	27.617	20.397
HM188398_DU	47.700	43.500	45.600	43.300	29.844	23.466	25.331	21.360	44.826	55.174	0.313	0.285	0.372	0.223	0.417	54.600	0.663	29.964	24.368	26.715	18.953
HM188399_DU	47.700	43.500	45.600	43.100	29.844	23.406	25.391	21.360	44.765	55.235	0.312	0.285	0.374	0.223	0.417	54.400	0.662	30.144	24.368	26.715	18.773

HM188400_DU	47.800	43.500	45.650	43.100	29.844	23.466	25.331	21.360	44.826	55.174	0.313	0.285	0.374	0.221	0.416	54.600	0.663	30.144	24.368	26.715	18.773
HM188401_DU	47.700	43.500	45.600	43.300	29.783	23.406	25.391	21.420	44.826	55.174	0.313	0.285	0.372	0.223	0.417	54.400	0.663	29.964	24.368	26.715	18.953
HM188402_DU	47.700	43.500	45.600	43.500	29.723	23.406	25.391	21.480	44.886	55.114	0.313	0.285	0.370	0.226	0.419	55.000	0.663	29.783	24.368	26.715	19.134
HQ317395_DU	47.700	43.900	45.800	43.500	29.783	23.586	25.211	21.420	45.006	54.994	0.317	0.283	0.366	0.228	0.419	54.800	0.665	29.422	24.188	27.076	19.314
HQ717357_DU	47.100	44.000	45.550	41.700	30.024	23.105	25.692	21.179	44.284	55.716	0.331	0.268	0.370	0.225	0.403	55.600	0.658	29.964	22.924	28.339	18.773
JN400895_DU	47.700	43.900	45.800	43.500	29.844	23.586	25.150	21.420	45.006	54.994	0.318	0.284	0.366	0.226	0.418	54.800	0.664	29.422	24.188	27.076	19.314
JQ013879_DU	47.100	43.500	45.300	43.000	29.904	23.225	25.572	21.300	44.525	55.475	0.318	0.280	0.368	0.228	0.416	55.600	0.660	29.783	24.007	27.256	18.953
KC750149_DU	47.700	42.800	45.250	41.200	30.024	22.744	26.113	21.119	43.863	56.137	0.342	0.265	0.373	0.218	0.394	53.900	0.653	29.783	22.563	29.061	18.592
KC750150_DU	47.700	43.000	45.350	43.700	28.881	22.563	26.354	22.202	44.765	55.235	0.335	0.267	0.345	0.249	0.421	56.300	0.657	27.617	22.924	28.700	20.758
KC750154_DU	46.800	43.500	45.150	42.600	29.483	22.503	26.233	21.781	44.284	55.716	0.339	0.263	0.354	0.243	0.411	55.700	0.657	28.520	22.383	28.881	20.217
KC750156_DU	47.700	43.300	45.500	43.500	29.783	23.345	25.391	21.480	44.826	55.174	0.312	0.285	0.370	0.228	0.420	54.600	0.663	29.783	24.368	26.715	19.134
KC750157_DU	47.500	43.000	45.250	40.600	30.205	22.623	26.113	21.059	43.682	56.318	0.345	0.260	0.375	0.217	0.388	53.900	0.651	30.144	22.022	29.242	18.592
KF219498_DU	47.500	42.800	45.150	41.000	30.144	22.684	26.113	21.059	43.742	56.258	0.343	0.264	0.372	0.216	0.392	54.200	0.651	29.964	22.383	29.061	18.592
KF771883_DU	46.900	43.000	44.950	43.700	30.024	23.345	25.451	21.179	44.525	55.475	0.312	0.285	0.369	0.232	0.422	55.700	0.658	29.603	24.368	26.715	19.314
KJ136259_DU	46.800	43.500	45.150	43.700	30.144	23.526	25.211	21.119	44.645	55.355	0.307	0.288	0.372	0.226	0.422	55.600	0.654	29.964	24.729	26.354	18.953
KJ525678_DU	47.500	43.900	45.700	43.000	29.783	23.345	25.451	21.420	44.765	55.235	0.324	0.277	0.366	0.228	0.414	54.700	0.663	29.422	23.646	27.617	19.314
KJ525679_DU	46.900	43.300	45.100	42.100	29.783	22.503	26.113	21.600	44.103	55.897	0.338	0.262	0.362	0.235	0.404	55.200	0.655	29.242	22.202	28.700	19.856
KJ525680_DU	47.100	42.800	44.950	43.000	30.024	22.924	25.692	21.360	44.284	55.716	0.325	0.276	0.366	0.234	0.415	55.800	0.657	29.422	23.466	27.617	19.495
KJ525681_DU	47.100	42.800	44.950	42.800	30.084	23.105	25.692	21.119	44.224	55.776	0.325	0.280	0.369	0.224	0.412	54.700	0.660	29.603	23.827	27.617	18.953
KJ525682_DU	47.100	43.000	45.050	41.900	30.205	22.984	25.812	20.999	43.983	56.017	0.328	0.273	0.375	0.220	0.403	54.000	0.660	30.144	23.285	27.978	18.592
KJ600786_DU	47.800	44.400	46.100	43.300	29.723	23.586	25.090	21.600	45.187	54.813	0.317	0.283	0.367	0.225	0.417	54.100	0.665	29.603	24.188	27.076	19.134
KT381594_DU	47.500	42.600	45.050	41.900	30.144	22.924	25.872	21.059	43.983	56.017	0.326	0.275	0.379	0.218	0.403	54.400	0.660	30.325	23.466	27.798	18.412
KU933948_DU	46.900	44.000	45.450	43.500	30.024	23.526	25.150	21.300	44.826	55.174	0.307	0.286	0.374	0.225	0.420	55.200	0.660	30.144	24.549	26.354	18.953
KU933949_DU	47.100	43.300	45.200	43.500	30.024	23.406	25.331	21.239	44.645	55.355	0.310	0.286	0.372	0.226	0.420	55.300	0.652	29.964	24.549	26.534	18.953
KU933950_DU	46.900	43.900	45.400	43.000	30.144	23.406	25.271	21.179	44.585	55.415	0.309	0.284	0.377	0.223	0.416	54.000	0.659	30.505	24.368	26.534	18.592
KU933951_DU	47.100	43.900	45.500	43.700	29.904	23.586	25.211	21.300	44.886	55.114	0.307	0.290	0.370	0.225	0.423	54.800	0.665	29.964	24.910	26.354	18.773
KX765177_DU	47.300	43.500	45.400	43.700	29.663	23.105	25.511	21.721	44.826	55.174	0.322	0.275	0.357	0.243	0.422	55.400	0.660	28.881	23.466	27.437	20.217
KX765178_DU	46.900	43.700	45.300	43.500	29.783	23.165	25.511	21.540	44.705	55.295	0.321	0.277	0.360	0.238	0.420	55.500	0.660	29.061	23.646	27.437	19.856
MF278932_DU	47.500	43.000	45.250	41.000	30.265	22.864	25.933	20.939	43.803	56.197	0.340	0.265	0.375	0.217	0.393	53.700	0.652	30.144	22.563	28.881	18.412
MH105249_DU	46.000	43.700	44.850	43.700	29.663	22.924	25.872	21.540	44.465	55.535	0.324	0.267	0.354	0.253	0.423	58.100	0.662	28.520	22.924	27.798	20.758
MH105250_DU	47.300	43.500	45.400	42.100	29.663	22.563	26.053	21.721	44.284	55.716	0.337	0.263	0.362	0.235	0.406	55.000	0.656	29.242	22.383	28.700	19.675
NC039223t_DU	47.300	44.600	45.950	43.500	29.483	23.285	25.391	21.841	45.126	54.874	0.317	0.277	0.363	0.235	0.420	54.700	0.658	29.242	23.827	27.256	19.675
AF162714_GO	47.300	43.500	45.400	43.100	29.483	22.864	25.872	21.781	44.645	55.355	0.328	0.267	0.357	0.243	0.417	55.800	0.653	28.700	22.924	28.159	20.217
AF431744_GO	47.700	43.700	45.700	44.600	29.242	23.225	25.451	22.082	45.307	54.693	0.318	0.278	0.348	0.249	0.431	58.900	0.645	28.159	23.827	27.256	20.758
AF456438_GO	47.500	43.300	45.400	44.200	29.422	23.105	25.572	21.901	45.006	54.994	0.318	0.278	0.354	0.246	0.428	56.400	0.648	28.520	23.827	27.256	20.397
AF456442_GO	47.100	43.700	45.400	43.900	29.483	23.105	25.632	21.781	44.886	55.114	0.326	0.275	0.351	0.243	0.423	55.300	0.656	28.339	23.466	27.798	20.397
AF473851_GO	47.300	43.700	45.500	43.100	29.543	22.924	25.752	21.781	44.705	55.295	0.333	0.267	0.353	0.245	0.417	56.100	0.647	28.520	22.744	28.339	20.397
DQ227246_GO	47.300	43.100	45.200	44.000	29.603	23.285	25.572	21.540	44.826	55.174	0.321	0.283	0.354	0.242	0.427	56.200	0.650	28.520	24.188	27.437	19.856
DQ363531_GO	47.700	43.300	45.500	43.900	29.483	23.045	25.572	21.901	44.946	55.054	0.322	0.273	0.353	0.249	0.425	56.600	0.649	28.700	23.285	27.437	20.578
DQ363534_GO	46.900	43.000	44.950	42.400	29.723	22.443	26.173	21.661	44.103	55.897	0.338	0.263	0.358	0.240	0.409	56.300	0.650	28.881	22.383	28.700	20.036
DQ417110_GO	46.900	43.100	45.000	42.600	29.663	22.503	26.113	21.721	44.224	55.776	0.338	0.264	0.357	0.240	0.410	56.200	0.651	28.700	22.383	28.700	20.217
DQ659677_GO	47.500	43.300	45.400	43.100	29.603	22.984	25.752	21.661	44.645	55.355	0.327	0.272	0.359	0.239	0.417	54.600	0.660	28.881	23.285	27.978	19.856
DQ858357_GO	47.300	42.800	45.050	44.200	29.663	23.225	25.572	21.540	44.765	55.235	0.318	0.284	0.357	0.243	0.428	56.100	0.649	28.700	24.188	27.076	20.036
EF579734_GO	46.800	43.100	44.950	42.800	29.603	22.503	26.173	21.721	44.224	55.776	0.338	0.264	0.355	0.243	0.412	56.200	0.651	28.520	22.383	28.700	20.397

FJ240168_GO	46.900	43.700	45.300	42.600	29.603	22.744	25.993	21.661	44.404	55.596	0.334	0.269	0.358	0.235	0.411	55.300	0.655	28.881	22.924	28.520	19.675
FJ480822_GO	47.800	43.500	45.650	43.100	29.543	23.165	25.632	21.661	44.826	55.174	0.326	0.273	0.359	0.238	0.418	54.700	0.658	28.881	23.466	27.978	19.675
FJ754273_GO	47.100	43.900	45.500	43.300	29.483	22.924	25.752	21.841	44.765	55.235	0.329	0.268	0.354	0.246	0.419	56.100	0.650	28.520	22.924	28.159	20.397
FS25_GO	47.300	42.600	44.950	42.400	29.904	22.864	25.993	21.239	44.103	55.897	0.331	0.273	0.368	0.228	0.408	54.700	0.662	29.422	23.285	28.159	19.134
GQ245793_GO	46.800	43.500	45.150	43.900	29.603	23.045	25.692	21.661	44.705	55.295	0.325	0.274	0.352	0.250	0.425	54.900	0.654	28.339	23.466	27.798	20.397
GQ245794_GO	46.600	43.500	45.050	44.000	29.483	23.045	25.812	21.661	44.705	55.295	0.324	0.274	0.350	0.253	0.427	55.300	0.652	28.159	23.466	27.798	20.578
GQ245800_GO	46.200	43.900	45.050	43.000	30.084	23.165	25.572	21.179	44.344	55.656	0.319	0.279	0.368	0.229	0.415	54.000	0.664	29.783	23.827	27.256	19.134
GQ255639_GO	47.500	43.700	45.600	44.000	29.362	23.105	25.572	21.961	45.066	54.934	0.320	0.276	0.353	0.244	0.426	56.300	0.648	28.520	23.646	27.437	20.397
JF340367_GO	47.300	43.900	45.600	43.300	29.723	23.225	25.451	21.600	44.826	55.174	0.321	0.277	0.362	0.235	0.419	55.700	0.649	29.242	23.646	27.437	19.675
JN631747_GO	47.100	43.500	45.300	43.100	30.024	23.285	25.391	21.300	44.585	55.415	0.312	0.283	0.373	0.225	0.417	54.800	0.657	30.144	24.188	26.715	18.953
JQ013862_GO	47.100	43.300	45.200	42.200	29.723	22.684	26.053	21.540	44.224	55.776	0.332	0.264	0.365	0.235	0.407	55.600	0.652	29.422	22.563	28.339	19.675
KJ136260_GO	46.600	43.500	45.050	43.100	30.205	23.285	25.391	21.119	44.404	55.596	0.310	0.284	0.376	0.224	0.417	53.400	0.655	30.325	24.368	26.534	18.773
KJ525676_GO	46.900	43.100	45.000	42.600	29.603	22.503	26.173	21.721	44.224	55.776	0.339	0.262	0.357	0.243	0.410	56.200	0.651	28.700	22.202	28.700	20.397
KJ525677_GO	46.900	42.800	44.850	42.200	30.205	23.045	25.812	20.939	43.983	56.017	0.326	0.278	0.373	0.220	0.406	54.100	0.658	29.964	23.646	27.798	18.592
KJ528559_GO	47.500	43.300	45.400	43.300	29.543	23.045	25.752	21.661	44.705	55.295	0.327	0.272	0.357	0.241	0.419	54.600	0.660	28.700	23.285	27.978	20.036
KJ607169_GO	47.300	43.300	45.300	43.100	29.663	22.984	25.752	21.600	44.585	55.415	0.327	0.272	0.359	0.239	0.417	54.700	0.660	28.881	23.285	27.978	19.856
KJ782375_GO	47.300	43.300	45.300	43.100	29.543	22.864	25.872	21.721	44.585	55.415	0.329	0.268	0.357	0.243	0.417	55.400	0.654	28.700	22.924	28.159	20.217
KT760569_GO	46.200	43.300	44.750	43.100	29.543	22.623	26.233	21.600	44.224	55.776	0.335	0.261	0.350	0.255	0.418	54.600	0.664	28.159	22.383	28.700	20.758
KU200249_GO	47.500	43.700	45.600	43.700	29.362	22.864	25.692	22.082	44.946	55.054	0.331	0.268	0.348	0.251	0.422	57.200	0.646	28.159	22.744	28.159	20.939
KU200250_GO	47.300	43.500	45.400	43.700	29.603	22.984	25.572	21.841	44.826	55.174	0.328	0.271	0.350	0.249	0.423	56.100	0.648	28.339	23.105	27.978	20.578
KU200252_GO	46.800	43.000	44.900	43.300	29.723	22.924	25.933	21.420	44.344	55.656	0.323	0.274	0.361	0.239	0.419	55.100	0.650	29.061	23.466	27.617	19.856
MK124761_GO	47.500	43.700	45.600	44.000	29.362	23.105	25.572	21.961	45.066	54.934	0.320	0.276	0.353	0.244	0.426	56.300	0.648	28.520	23.646	27.437	20.397
ZHE_GO	47.500	42.600	45.050	41.900	30.144	22.924	25.872	21.059	43.983	56.017	0.326	0.275	0.379	0.218	0.403	54.400	0.657	30.325	23.466	27.798	18.412

Table S3. The RSCU value of 59 codons encoding 19 amino acids according to two hosts of NDV VII F gene. The preferred synonymous codons are shown in bold.

Amino acid	Codon	Host			Amino acid	Codon	Host		
		Chicken	Duck	Goose			Chicken	Duck	Goose
Phe	UUU(F)	**1.15**	**1.12**	**1.18**	Ala	GCU(A)	0.87	0.9	0.88
	UUC(F)	0.85	0.88	0.82		GCC(A)	0.98	0.97	0.97
Leu	UUA(L)	**1.22**	**1.2**	**1.18**		GCA(A)	**1.73**	**1.73**	**1.66**
	UUG(L)	0.93	0.9	1		GCG(A)	0.41	0.41	0.49
	CUU(L)	1.07	1.08	1.12	Tyr	UAU(Y)	**1.21**	**1.27**	**1.25**
	CUC(L)	0.83	0.84	0.83		UAC(Y)	0.79	0.73	0.75
	CUA(L)	0.84	0.85	0.82	His	CAU(H)	**1.94**	**2**	**1.95**
	CUG(L)	1.1	1.12	1.06		CAC(H)	0.06	0	0.05
Ile	AUU(I)	0.77	0.76	0.74	Gln	CAA(Q)	**1.01**	**1.03**	**1.03**
	AUC(I)	1	1.02	1.02		CAG(Q)	0.99	0.97	0.97
	AUA(I)	**1.24**	**1.22**	**1.24**	Asn	AAU(N)	**1.35**	**1.36**	**1.35**
Val	GUU(V)	0.71	0.68	0.76		AAC(N)	0.65	0.64	0.65
	GUC(V)	**1.45**	**1.47**	**1.4**	Lys	AAA(K)	0.81	0.83	0.8
	GUA(V)	1.07	1.06	1		AAG(K)	**1.19**	**1.17**	**1.2**
	GUG(V)	0.78	0.79	0.84	Asp	GAU(D)	**1.1**	**1.11**	**1.1**
Ser	UCU(S)	1.07	1.11	1.24		GAC(D)	0.9	0.89	0.9
	UCC(S)	1.11	1.06	0.96	Glu	GAA(E)	**1.32**	**1.3**	**1.32**
	UCA(S)	**1.85**	**1.87**	**1.83**		GAG(E)	0.68	0.7	0.68
	UCG(S)	0.33	0.32	0.36	Cys	UGU(C)	**1.47**	**1.58**	**1.62**
	AGU(S)	0.52	0.5	0.5		UGC(C)	0.53	0.42	0.38
	AGC(S)	1.13	1.15	1.11	Trp	UGG(W)	1	1	1
Pro	CCU(P)	**1.57**	**1.54**	**1.65**	Arg	CGU(R)	0.58	0.6	0.51
	CCC(P)	0.83	0.85	0.72		CGC(R)	0.51	0.51	0.58
	CCA(P)	0.98	0.97	0.92		CGA(R)	0.31	0.29	0.32
	CCG(P)	0.62	0.64	0.71		CGG(R)	0.55	0.55	0.61
Thr	ACU(T)	**1.35**	**1.35**	1.32		AGA(R)	**2.61**	**2.58**	**2.39**
	ACC(T)	1.05	1.09	1.08		AGG(R)	1.43	1.46	1.59
	ACA(T)	1.32	**1.35**	**1.39**	Gly	GGU(G)	1.05	1.08	1.06
	ACG(T)	0.28	0.21	0.21		GGC(G)	**1.13**	**1.11**	**1.14**
						GGA(G)	0.94	0.97	0.83
						GGG(G)	0.88	0.84	0.97
